# Supplementary material for: Differential leaf flooding resilience in Arabidopsis thaliana is controlled by ethylene signaling-activated and age-dependent phosphorylation of ORESARA1
Source: Plant Commun. 2024 Feb 19;5(6):100848. doi: 10.1016/j.xplc.2024.100848 (PMC11211547; doi:10.1016/j.xplc.2024.100848)
Supplement: Document S2. Article plus supplemental information [file mmc8.pdf]

Plant Communications
Research article

CellPress Partner Journal

Differential leaf flooding resilience in *Arabidopsis thaliana* is controlled by ethylene signaling-activated and age-dependent phosphorylation of ORESARA1

Tom Rankenberg[1], Hans van Veen[1,2], Mastoureh Sedaghatmehr[3], Che-Yang Liao[4], Muthanna Biddanda Devaiah[4], Evelien A. Stouten[1], Salma Balazadeh[5] and Rashmi Sasidharan[1,*]

[1]Plant Stress Resilience, Utrecht University, Padualaan 8, 3584 CH Utrecht, the Netherlands

[2]Evolutionary Plant-Ecophysiology, Groningen Institute for Evolutionary LIfe Sciences, Nijenborgh 7, 9747 AG Groningen, the Netherlands

[3]Max Planck Institute of Molecular Plant Physiology, Am Mühlenberg 1, 14476 Potsdam, Germany

[4]Experimental and Computational Plant Development, Utrecht University, Padualaan 8, 3584 CH Utrecht, the Netherlands

[5]Leiden University, Leiden, the Netherlands

***Correspondence: Rashmi Sasidharan (r.sasidharan@uu.nl)**

https://doi.org/10.1016/j.xplc.2024.100848

ABSTRACT

The phytohormone ethylene is a major regulator of plant adaptive responses to flooding. In flooded plant tissues, ethylene quickly increases to high concentrations owing to its low solubility and diffusion rates in water. Ethylene accumulation in submerged plant tissues makes it a reliable cue for triggering flood acclimation responses, including metabolic adjustments to cope with flood-induced hypoxia. However, persistent ethylene accumulation also accelerates leaf senescence. Stress-induced senescence hampers photosynthetic capacity and stress recovery. In submerged *Arabidopsis*, senescence follows a strict age-dependent pattern starting with the older leaves. Although mechanisms underlying ethylene-mediated senescence have been uncovered, it is unclear how submerged plants avoid indiscriminate breakdown of leaves despite high systemic ethylene accumulation. We demonstrate that although submergence triggers leaf-age-independent activation of ethylene signaling via EIN3 in *Arabidopsis*, senescence is initiated only in old leaves. EIN3 stabilization also leads to overall transcript and protein accumulation of the senescence-promoting transcription factor ORESARA1 (ORE1) in both old and young leaves during submergence. However, leaf-age-dependent senescence can be explained by ORE1 protein activation via phosphorylation specifically in old leaves, independent of the previously identified age-dependent control of *ORE1* via miR164. A systematic analysis of the roles of the major flooding stress cues and signaling pathways shows that only the combination of ethylene and darkness is sufficient to mimic submergence-induced senescence involving ORE1 accumulation and phosphorylation. Hypoxia, most often associated with flooding stress in plants, appears to have no role in these processes. Our results reveal a mechanism by which plants regulate the speed and pattern of senescence during environmental stresses such as flooding. Age-dependent ORE1 activity ensures that older, expendable leaves are dismantled first, thus prolonging the life of younger leaves and meristematic tissues that are vital to whole-plant survival.

Key words: flooding, abiotic stress, hypoxia, senescence, ethylene

Rankenberg T., van Veen H., Sedaghatmehr M., Liao C.-Y., Devaiah M.B., Stouten E.A., Balazadeh S., and Sasidharan R. (2024). Differential leaf flooding resilience in *Arabidopsis thaliana* is controlled by ethylene signaling-activated and age-dependent phosphorylation of ORESARA1. Plant Comm. **5**, 100848.

INTRODUCTION

Ethylene is a gaseous hormone that controls many aspects of plant development and acts as a central regulator of plant envi-

Published by the Plant Communications Shanghai Editorial Office in association with Cell Press, an imprint of Elsevier Inc., on behalf of CSPB and CEMPS, CAS.

This is an open access article under the CC BY license (http://creativecommons.org/licenses/by/4.0/).

ronmental stress responses (Sasidharan and Voesenek, 2015; Dubois et al., 2018; Leeggangers et al., 2023). Plant endogenous ethylene concentrations increase in response to a wide variety of abiotic stresses, primarily mediated by enhanced ethylene biosynthesis (Argueso et al., 2007). Subsequently, ethylene triggers stabilization of the key transcription factor ETHYLENE-INSENSITIVE1 (EIN3), leading to downstream transcriptional cascades that culminate in various stress responses (Chang et al., 2013; Binder, 2020).

Flooded plants present an exception to the stress-mediated increase in ethylene biosynthesis. At least immediately following flooding, ethylene levels increase rapidly in submerged plant tissues owing to physical entrapment by the surrounding flood water. This quick increase in gaseous ethylene to physiologically saturating concentrations is a consequence of severely limited gas diffusion underwater (Voesenek and Sasidharan, 2013; Xie et al., 2015; Hartman et al., 2019). Ethylene accumulation and consequent stabilization of EIN3 is used by plants as an early flooding signal. Ethylene is a major regulator of flood-adaptive traits and influences performance during flooding in various ways. For example, ethylene signaling induces stem elongation during submergence in deepwater rice by inducing gibberellin biosynthesis and signaling (Métraux and Kende, 1983; Hattori et al., 2009; Kuroha et al., 2018). In lowland rice, on the other hand, ethylene signaling represses shoot elongation and carbohydrate consumption via SUB1A (Fukao et al., 2006; Xu et al., 2006; Fukao and Bailey-Serres, 2008). In *Arabidopsis thaliana* (Arabidopsis), ethylene signaling aids transcriptional responses to flood-induced tissue hypoxia, inhibits growth, and modulates damage caused by reactive oxygen species to enhance hypoxia survival (Peng et al., 2001; Tsai et al., 2014; Hartman et al., 2019; Liu et al., 2022).

Characterization of ethylene functions in plant flooding responses has focused primarily on traits that aid survival. However, considering its well-established role as a positive regulator of senescence (Graham et al., 2012), ethylene accumulation likely accelerates leaf senescence during submergence. Leaf senescence is often considered a marker for flood sensitivity, as flooding-intolerant accessions of rice, Arabidopsis, maize, and *Lotus japonicus* display more severe leaf senescence during flooding and post-flooding compared with tolerant accessions (Krishnan et al., 1999; Campbell et al., 2015; Alpuerto et al., 2016; Yeung et al., 2018; Buraschi et al., 2020). Furthermore, Arabidopsis mutants with reduced senescence exhibit improved performance after submergence compared with wild-type plants (Zhang et al., 1997; Yeung et al., 2018).

The response of plant tissues to ethylene strongly depends on tissue age (Doubt, 1917; Chen et al., 2013; Ceusters and Van de Poel, 2018). Ethylene treatment induces senescence much faster in older leaves than in younger leaves (dela Fuente and Leopold, 1968; Jing et al., 2005). This ensures that senescence and death occur only when a leaf has reached maturity. Some mechanisms that contribute to this age-dependent response to ethylene have been identified. As a leaf ages, *EIN3* transcription gradually increases, intensifying the strength of the response to endogenous ethylene (Li et al., 2013). EIN3 induces transcription of the master senescence regulator *ORESARA1* (*ORE1*), a NAC transcription factor whose activity is controlled by the kinase CALCIUM-DEPENDENT PROTEIN KINASE1 (CPK1) (Durian et al., 2020). However, premature senescence is prevented in young leaves through the degradation of *ORE1* mRNA by the microRNA *miR164* (Kim et al., 2009). As a leaf ages, the abundance of *miR164* decreases, which leads to a gradual accumulation of *ORE1*. This gradient in *miR164* works as a buffer that prevents untimely senescence in young leaves. However, increased ethylene production in stressed plants can accelerate senescence even in young leaves.

In submerged Arabidopsis rosettes that would experience systemic accumulation of ethylene, senescence still occurs along a strict leaf-age-dependent gradient. Here, we investigated the mechanisms underlying this sequential leaf death. We first established that this pattern was dependent on ethylene sensing but did not require hypoxia sensing via the N-degron pathway, which is another important signaling cascade for flood acclimation. Next, we found that ethylene signaling is activated in a leaf-age-independent manner upon submergence and via EIN3, which induces accumulation of the senescence-regulating transcription factor ORESARA1 (ORE1), indicating a *miR164*-independent mechanism. Although ORE1 protein was present in old and young leaves during submergence, ORE1 activation and senescence were triggered only in old leaves owing to age-dependent phosphorylation of ORE1 in these tissues, independent of *CPK1*. This age-dependent phosphorylation of ORE1 ensures that leaf senescence during flooding follows an age-dependent gradient, preventing systemic tissue degradation and prolonging shoot survival.

RESULTS

Ethylene perception during submergence is systemic but mediates age-dependent leaf death

Arabidopsis plants (10-leaf stage) that were completely submerged in the dark (hereafter "submerged" unless otherwise specified) for varying durations exhibited a typical age-dependent pattern of leaf death. This sequential leaf death started in the oldest leaves and progressed down the age gradient toward the youngest leaves and shoot apex, which died last (Figure 1A and Supplemental Video 1). Ethylene has been identified as an important regulator of both flooding responses and age-dependent stress responses (Sasidharan and Voesenek, 2015; Ceusters and Van de Poel, 2018; Rankenberg et al., 2021). It has previously been established that ethylene accumulates quickly in flooded tissues (Sasidharan and Voesenek, 2015), resulting in rapid stabilization of EIN3 (Xie et al., 2015; Hartman et al., 2019). EIN3 is a transcription factor that acts as a key regulator of downstream transcriptional responses to ethylene (Chao et al., 1997; Chang et al., 2013). However, considering that leaf death was not triggered uniformly across the submerged Arabidopsis rosettes, we wanted to establish whether ethylene signaling was indeed systemic. For this, we monitored levels of EIN3 protein in old (leaf 3) and young (leaf 7) leaves. Submergence enhanced EIN3 levels in both old and young leaves within a few hours, consistent with the expected rapid accumulation of ethylene (Figure 1B). Interestingly, although EIN3 was stabilized rapidly following submergence, levels decreased thereafter during the first 24 h of submergence. This is in agreement with previous observations of EIN3 as a hit-and-run transcription factor, binding briefly to its downstream targets, after which their transcription is maintained

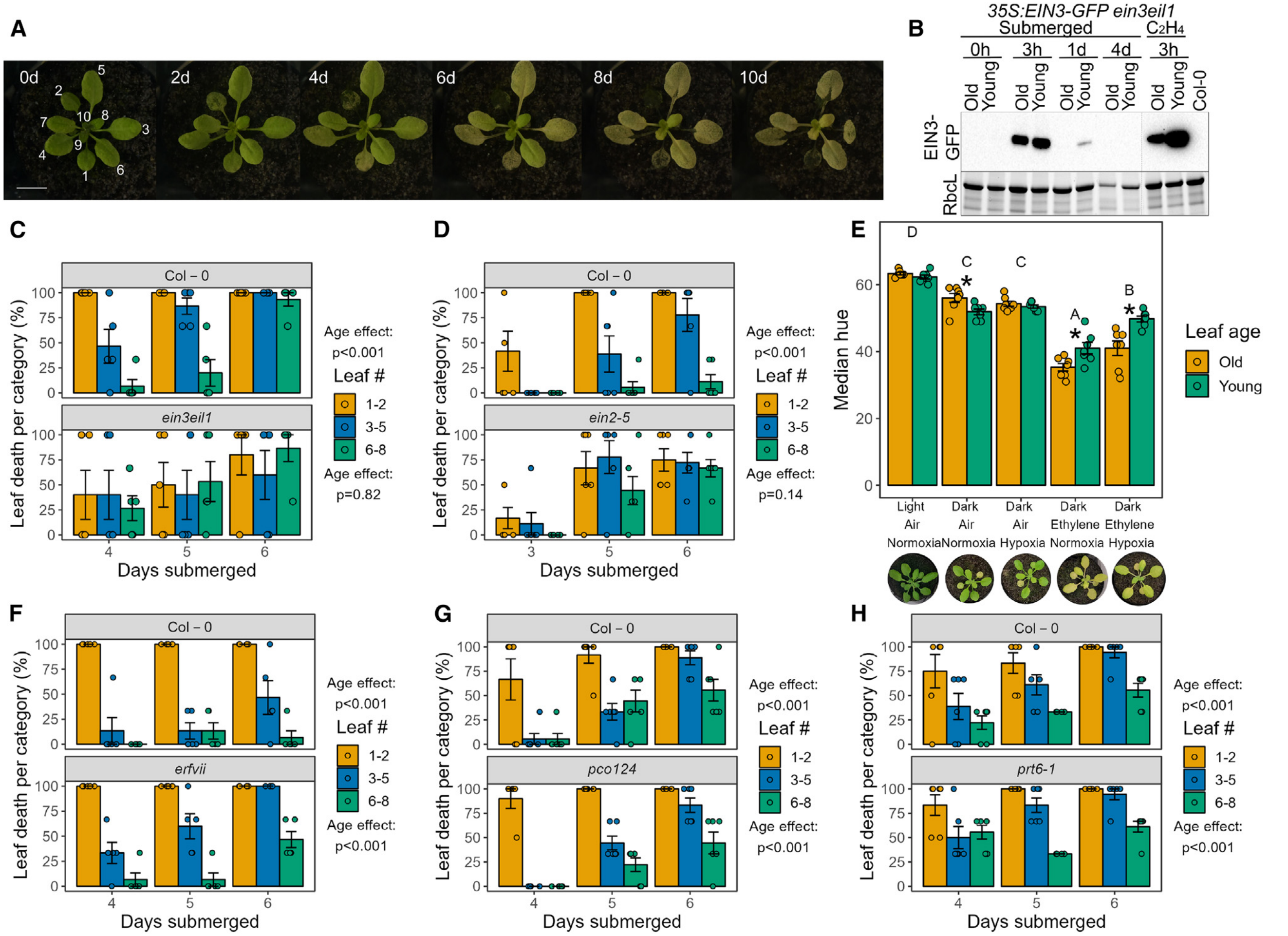

Figure 1. Ethylene perception during submergence is systemic but mediates age-dependent leaf death

(A) Arabidopsis wild-type (accession Col-0) plants submerged in darkness show age-dependent leaf death. Images show representative plants submerged for the duration indicated in the top left of each image. Numbers in the first image indicate leaf numbering according to age. Scale bar corresponds to 1 cm.

(B) Immunoblot analyses showing that EIN3-GFP accumulates within 3 h of submergence or ethylene treatment in both old (leaf 3) and young (leaf 7) leaves of transgenic *35S:EIN3-GFP ein3eil1* plants. Samples were run on the same gel; the vertical line indicates where samples were cropped out. The large subunit of Rubisco (RbcL) served as a loading control.

(C and D) Quantification of leaf death across three age categories of an Arabidopsis rosette. Age categories are indicated by leaf number (#) as in **(A)**. Age-dependent leaf death observed in wild-type plants is lost in ethylene-insensitive *ein3eil1* and *ein2-5* mutants. *P* values indicate the effect of leaf age on the proportion of dead leaves per genotype, determined by a two-way ANOVA (leaf age × submergence duration). n = 5–6 plants per time point.

(E) The effect of different flooding cues on leaf yellowing in wild-type Arabidopsis plants. Yellowing is indicated by the median hue of old (leaf 3) and young (leaf 7) leaves after 4 days of exposure to each treatment. Ethylene induces age-dependent leaf yellowing, and this process is slowed by hypoxia. Images below each bar show representative plants from each treatment. Asterisks indicate differences between old (leaf 3) and young (leaf 7) leaves (paired t-test), and different letters indicate significant differences between treatments (two-way ANOVA and Tukey's post-hoc test). n = 7 plants per treatment. Treatment combinations are indicated, where normal (normoxia) or low oxygen (hypoxia) was combined with (ethylene) or without (air) ethylene gas in the presence (light) or absence (dark) of light.

(F–H) Age-dependent leaf death is not lost in *pco124*, *erfVII*, or *prt6-1* mutants, which have impaired oxygen sensing. Age categories are indicated by leaf number (#) as in **(A)**. *P* values indicate the effect of leaf age on the proportion of dead leaves per genotype determined by a two-way ANOVA (leaf age × submergence duration). n = 5–6 plants per time point.

by other regulators (Chang et al., 2013; Alvarez et al., 2021). After establishing that ethylene signaling was activated systemically in flooded Arabidopsis plants, we next investigated the role of ethylene in the observed leaf-age-dependent senescence gradient. Age-dependent leaf death was quantified by dividing leaves into three categories based on their order of emergence: leaves 1 and 2, 3 to 5, and 6 to 8. Leaves were scored as dead when more than half of their blade area had desiccated after 3 days of post-submergence recovery, and the proportion of dead leaves per category was calculated for each plant. This confirmed a significant leaf-age effect in wild-type plants (Figure 1C). However, this was lost in the ethylene-insensitive *ein3eil1* and *ein2-5* mutants (Figure 1C and 1D and supplemental Figure 1B), indicating the involvement of ethylene signaling. We observed some variation between experiments in the speed at which leaves of submerged plants died. However, in all experiments, a

gradient in leaf death with age was consistently observed in plants that could respond to ethylene. In addition to ethylene accumulation, submergence especially during a light-limited flooding event also causes a significant decline in tissue oxygen levels (Vashisht et al., 2011; Sasidharan et al., 2018). To further probe the relative importance of ethylene in regulating the observed pattern of leaf death during submergence, we exposed plants to combinations of the main submergence signals—ethylene, darkness, and hypoxia. The median hue of representative old (leaf 3) and young (leaf 7) leaves was used to quantify yellowing. The combination of ethylene and darkness induced age-dependent leaf yellowing, and adding hypoxia to this combination ameliorated it (Figure 1E). However, hypoxia or darkness alone failed to trigger sequential leaf yellowing. Considering the established importance of hypoxia as a flooding stress cue, we tested whether submergence-induced sequential leaf death requires hypoxia sensing. For this, we used the Arabidopsis mutants *erfVII*, *pco124*, and *prt6-1*, which lack important components of the plant oxygen-sensing machinery (Abbas et al., 2015; Masson et al., 2019). In all these mutants, submergence still triggered the sequential leaf-death pattern observed in the wild type, suggesting that this response does not require oxygen sensing via the N-degron pathway (Figure 1F–1H and Supplemental Figure 1B).

In conclusion, we confirmed that submergence-mediated sequential leaf death requires ethylene signaling. Also, despite systemic activation of ethylene signaling in submerged rosettes, leaf death occurs in a more localized, defined pattern. The mechanisms underlying this observation were of interest for further study.

Submergence-induced senescence is primarily controlled by the ethylene-responsive NAC-domain transcription factor ORE1

The regulatory networks that underpin ethylene-mediated chlorophyll degradation leading to leaf senescence and death are well established (Woo et al., 2019). Relevant to submergence-induced senescence is the activation by ethylene of the NAC domain transcription factor ORE1 (Qiu et al., 2015; Yeung et al., 2018). ORE1 is a positive regulator of leaf senescence. Because the function of the EIN3–ORE1 regulon during leaf senescence is well established, we used this as a system to investigate how ethylene-mediated leaf senescence is coordinated in an age-dependent manner during submergence (Kim et al., 2009; Li et al., 2013; Qiu et al., 2015).

Consistent with previous reports, ethylene-mediated senescence was reduced in *ore1-1* knockout mutants (Figure 2A) (Li et al., 2013; Qiu et al., 2015), and ethylene exposure triggered a substantial increase in *ORE1* transcripts (Figure 2B). Interestingly, this increase was observed in both old and young leaves.

Next, we set out to establish that ORE1 is indeed a principal regulator of submergence-induced senescence. Consistent with the role of ORE1 as a positive regulator of senescence, submergence-induced senescence was significantly reduced and enhanced in *ore1* mutants and overexpressors, respectively (Figure 2C and 2D, Supplemental Figure 2A–2C, and Supplemental Video 2). Moreover, the higher chlorophyll retention phenotype of *ore1-1* mutants during submergence could be reverted to the wild-type phenotype by complementation with *ORE1* (ORE1 fused to an HA tag driven by its own promoter) (Figure 2E). ORE1 plays a role in dark-induced senescence of detached leaves (Kim et al., 2018). We did not detect visual signs of senescence in whole plants treated with darkness for the experimental duration used here (Figure 1D and Supplemental Figure 2D), and the effect of darkness on rosette area did not differ between Col-0 and *ore1-1* (Supplemental Figure 2E). These results show that the submergence phenotype of *ore1-1* mutants is not merely an effect of darkness. In general, higher chlorophyll maintenance in *ore1-1* mutants corresponded with better performance during submergence relative to the wild type. This was reflected in a smaller number of dead leaves and lower electrolyte leakage, although there were no significant differences in the rate of new leaf initiation immediately following desubmergence (Figure 2F and Supplemental Figure 2F and 2G). However, submerged *ore1-1* mutants also displayed a greater retention of healthy rosette area, which led to a greater rosette area after prolonged recovery (supplemental Figure 2H and 2H), and *ore1-1* seed yield was not compromised by flooding (Figure 2I). Under control conditions, *ore1-1* mutants did show a significant reduction in seed yield compared with wild-type plants. This can be attributed to delayed leaf senescence in *ore1-1* mutants. Leaf senescence plays a vital role in remobilizing nutrients from dying leaves for seed production at the end of a plant's lifecycle (Havé et al., 2017).

Notably, we found that the leaf phenotype of *ore1* mutants was age dependent: the reduction in leaf senescence during flooding was most visible in old leaves (Figure 2C and Supplemental Figure 2A). Consistent with this visual observation, the decrease in chlorophyll content and cell membrane integrity was greatest in old leaves of Col-0 plants (Figure 2D and 2G). We thus set out to investigate the regulation of ORE1 and determine how it is activated in an age-dependent manner.

Leaf-age-dependent regulation of ORE1

Submergence strongly enhanced *ORE1* transcript levels in whole rosettes, and this effect was also maintained during recovery. Although darkness also triggered upregulation of *ORE1*, levels quickly dropped as plants were placed back in the light (Figure 3A).

Surprisingly, submergence led to increased transcript levels of *ORE1* in both old and young leaves (Figure 3B). This was confirmed using a transgenic line in which the 1.6-kb promoter of *ORE1* was fused to a GUS enzyme. GUS staining patterns in both young and old leaves confirmed age-independent *ORE1* promoter activity during flooding (Supplemental Figure 3A). Next, we examined whether age-dependent differences in *ORE1* occurred at the protein level. To do so, we complemented the *ore1-1* mutant line with an HA-tagged version of ORE1 driven by its native 1.6-kb promoter. In the *pORE1:ORE1-HA ore1-1* lines, there was an accumulation of ORE1 protein in both old and young leaves at both 1 and 3 days of submergence (Figure 3C). We did not detect any ORE1 protein in either old or young leaves of non-submerged plants, as short-day-grown Arabidopsis plants at the 10-leaf stage have not yet initiated senescence of their oldest leaves. Although *ORE1* protein and mRNA accumulated in both old and young leaves during

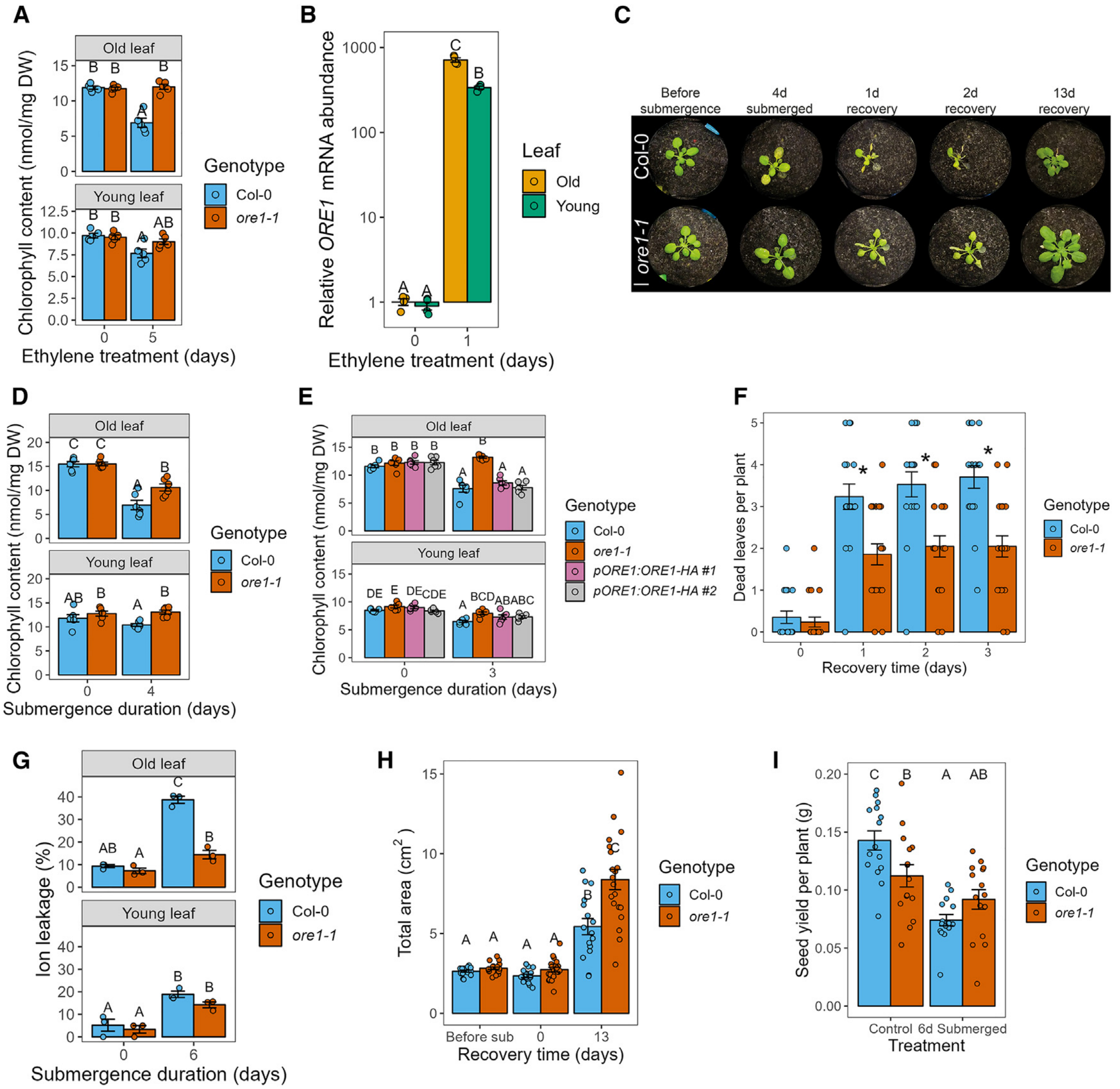

Figure 2. Submergence-induced senescence is primarily controlled by the ethylene-responsive NAC transcription factor ORE1
(A) Chlorophyll content of old and young leaves of Col-0 and *ore1-1* plants before and after 5 days of ethylene treatment. *n* = 5 leaves per sample.
(B) *ORE1* mRNA abundance increases in both old and young leaves after 1 day of ethylene treatment. *n* = 4 leaves per sample, each consisting of 2 old or young leaves from different plants pooled together. Expression levels were normalized to those in old leaves of non-submerged plants.
(C) *ore1-1* mutants show reduced yellowing of old leaves after 4 days of submergence. Representative images show Col-0 and *ore1-1* plants at the indicated time points. Scale bar corresponds to 1 cm.
(D) Chlorophyll content of old and young leaves of Col-0 and *ore1-1* before and after 4 days of submergence. *n* = 6 leaves per sample.
(E) Chlorophyll content of Col-0, *ore1-1*, and two independent *pORE1:ORE1-HA ore1-1* lines before and after 3 days of submergence. *n* = 6 leaves per sample.
(F) Dead leaves per Col-0 and *ore1-1* plant during recovery from 4 days of submergence. Leaves were scored as dead or alive at each of the indicated time points, *n* = 17–21 plants per genotype. These same plants were phenotyped for supplemental Figure 2E and 2H.
(G) Ion leakage of Col-0 and *ore1-1* before and after 6 days of submergence. *n* = 3 pools of 5 old or young leaves from different plants per sample.
(H) Total living rosette area of Col-0 and *ore1-1* plants before and after 4 days of submergence (sub) and after 13 days of recovery. Images of plants were categorized into dead, senescing, and healthy pixels using PlantCV. Senescing and healthy pixels were combined for each plant and converted to an area in cm^2. *n* = 16–20 plants per sample.
(I) Seed yield of Col-0 and *ore1-1* plants under control conditions and of plants that were submerged for 6 days. *n* = 15 plants per group. Different letters indicate significant differences between groups (two-way ANOVA and Tukey's post-hoc test). Asterisks indicate significant differences between Col-0 and *ore1-1* per time point.

submergence, mRNA of the ORE1 target *BIFUNCTIONAL NUCLEASE1* accumulated only in old leaves (Figure 3D). *ein3eil1* mutants still exhibited a modest increase in *ORE1* mRNA levels during submergence, but this did not lead to an increase in *BFN1* mRNA levels (Figure 3D).

Ethylene is known to enhance *ORE1* mRNA abundance via two routes—via direct transcriptional induction and via inhibition of its post-transcriptional repressor *miR164* (Kim et al., 2009). The latter mode is associated with age-dependent ethylene-induced senescence. Young leaves typically have high levels of *miR164,*

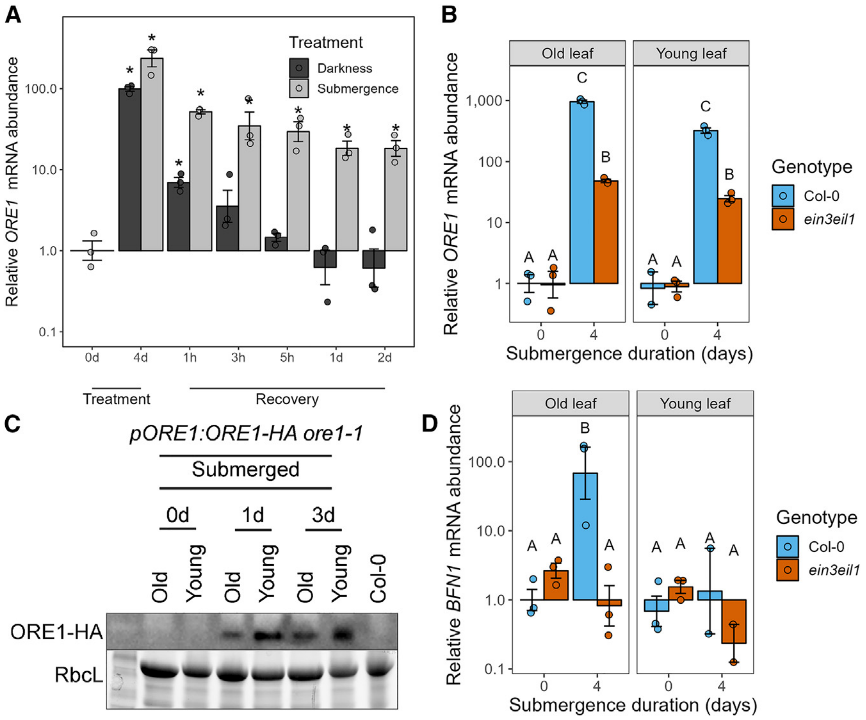

Figure 3. *ORE1* is induced in an age-independent manner during flooding stress

(A) *ORE1* mRNA abundance in whole rosettes before and after darkness and dark submergence. Asterisks indicate significant differences compared with untreated plants (one-way ANOVA and Dunnett's post-hoc test). Expression was normalized to that of untreated plants. $n = 3$, each sample consists of one rosette.

(B) *ORE1* mRNA abundance in old and young leaves of Col-0 and *ein3eil1* before and after 4 days of submergence. Different letters indicate significant differences between groups (two-way ANOVA and Tukey's post-hoc test). Expression was normalized to that of non-submerged old leaves of Col-0. Three biological replicates were analyzed. *ORE1* mRNA was not detected in one of the non-submerged Col-0 and *ein3eil1* young leaf samples; each sample consists of two leaves pooled together from different plants.

(C) Immunoblots showing ORE1-HA protein abundance in old and young leaves before and after 1 and 3 days of submergence using an antibody against HA. Each *pORE1:ORE1-HA ore1-1* sample consists of five old or young leaves pooled together from different plants. Proteins of the Col-0 sample were extracted from one whole rosette. The large subunit of Rubisco (RbcL) served as a loading control.

(D) mRNA abundance of the ORE1 target gene *BFN1* in old and young leaves of Col-0 and *ein3eil1* before and after 4 days of submergence. Different letters indicate significant differences between groups (two-way ANOVA and Tukey's post-hoc test). Expression was normalized to that of non-submerged old leaves of Col-0. Three biological replicates were analyzed. *BFN1* mRNA was not detected in one of the submerged Col-0 and *ein3eil1* young leaf samples; each sample consists of two leaves pooled together from different plants.

which decline with age. This ensures that *ORE1* mRNA is degraded when its transcription is induced by EIN3 and protects young leaves from premature senescence (Kim et al., 2009; Li et al., 2013). We observed significantly higher expression of *miR164b* in young leaves compared with old leaves (Supplemental Figure 3B), and although submergence caused a decline in *miR164b* abundance, age-specific differences were maintained. However, the similar accumulation of ORE1 protein in both old and young leaves (Figure 3C) suggests that degradation of *ORE1* mRNA by *miR164* is not sufficient to prevent premature accumulation of ORE1 protein during submergence.

Despite systemic ORE1 accumulation, downstream targets are activated in a leaf-age-dependent manner

Although ORE1 protein accumulated in old and young leaves during submergence (Figure 3C), *ORE1* knockout had a stronger effect on old leaves than on young leaves (Figure 2), and the ORE1 target *BFN1* was induced only in old leaves, suggesting that ORE1 activation occurs only in these tissues (Figure 3D). To strengthen this evidence and obtain a global and unbiased overview of whether there is age-dependent activation of ORE1 targets, we carried out an mRNA-seq experiment. Old and young leaves of Col-0 and *ore1-1* were harvested before submergence, after 4 days of submergence, and after 6 h of recovery (Figure 4A). Approximately 10 times as many differentially expressed genes (DEGs) were found between old leaves of Col-0 and *ore1-1* than between young leaves during submergence (Figure 4A). Interestingly, there were no genotype-specific DEGs when comparing the recovery time point with the pre-submergence time point. Of the 720 genotype-specific DEGs in the recovery vs. submergence comparison, 428 were already differentially expressed after 4 days of submergence. This suggests that *ORE1* knockout mostly affects the transcriptome of old leaves during submergence and not during recovery. Of the DEGs between old leaves of Col-0 and *ore1-1* during submergence, the subset that showed a smaller increase in expression during submergence in *ore1-1* than in Col-0 contained several previously identified targets of ORE1, including *BIFUNCTIONAL NUCLEASE1* (*BFN1*) and *NON-YELLOWING1* (*NYE1*). Furthermore, these DEGs were enriched for ORE1 binding sites near their transcriptional start sites (Supplemental Figure 4A). DEGs that did not fall within this subset did not have this enrichment, nor did non-DEGs.

We expanded the set of known ORE1 target genes by confirming that ORE1 can bind to the promoters of the protease *METACASPASE9* (*MC9*), the transcription factor *ANAC010*, the nuclease *DEFECTIVE IN POLLEN ORGANELLE DNA DEGRADATION 1* (*DPD1*), and the chloroplast-degrading protein *CHLOROPLAST VESICULATION* (*CV*) *in vitro* via electrophoretic mobility shift assay (EMSA) (Figure 4B). These new targets were selected on the basis of their roles in senescence-related processes. *In vivo* binding of ORE1 to these promoters was confirmed via ChIP–qPCR using 1-day-submerged *pORE1:ORE1-HA* plants (Figure 4C). Binding of ORE1 to all newly identified putative targets was significantly enriched when compared with the negative control (AT4G22180) (Figure 4C). Out of a set of 15 verified ORE1 targets from this and previous studies (Matallana-Ramirez et al., 2013; Rauf et al., 2013; Qiu et al., 2015; Zhang et al., 2021) (Supplemental Table 1), *ORE1*

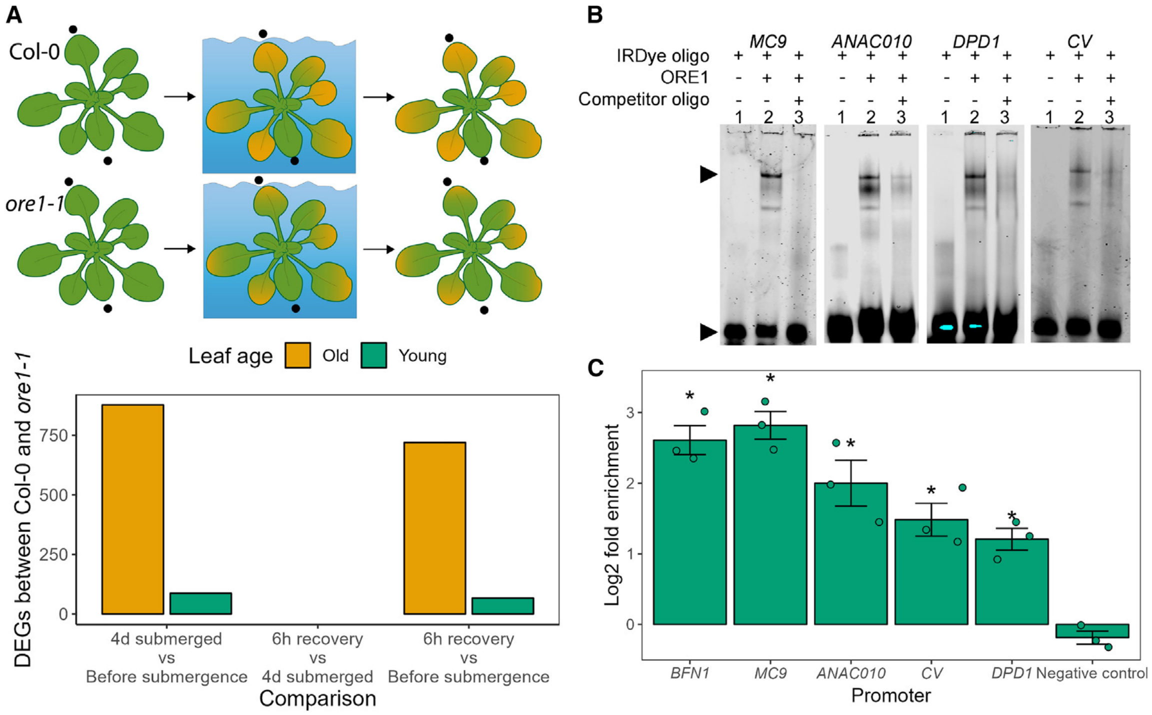

Figure 4. ORE1 target activation is age independent

(A) Leaf samples of Col-0 and *ore1-1* were harvested before submergence, after 4 days of submergence, and after 6 h of recovery. The number of differentially expressed genes (DEGs) that show a genotype-dependent response to 4 days of submergence is greater in old leaves than in young leaves. None showed a genotype-dependent effect in their response to submergence followed by recovery. Most (428/720) DEGs that showed a genotype-specific response to post-submergence recovery showed the opposite pattern during the submergence phase.

(B) Electrophoretic mobility shift assay (EMSA) showing *in vitro* binding of recombinant ORE1-GST to the promoters of *MC9*, *ANAC010*, *DPD1*, and *CV*. From left to right in each image: lane 1, labeled probe (5′-DY682-labeled double-stranded oligonucleotides); lane 2, labeled probe plus ORE1-GST protein; lane 3, labeled probe, ORE1-GST protein, and competitor (unlabeled oligonucleotide containing an ORE1 binding site; 200× molar access). Arrows indicate retarded bands (bound oligo) and non-bound DNA probes (free oligo).

(C) ChIP–qPCR showing *in vivo* binding of ORE1 to the promoters of *MC9*, *ANAC010*, *DPD1*, and *CV*. Asterisks indicate significant enrichment relative to the negative control (AT2G22180) (one-way ANOVA and Dunnett's post-hoc test). Chromatin was extracted from immunoprecipitated samples of whole *pORE1:ORE1-HA* rosettes submerged for 1 day, $n = 3$.

disruption affected the submergence induction of 12 (in old leaves). In young leaves, however, only 3 out of the 15 differed in their response to submergence between Col-0 and *ore1-1* (Supplemental Figure 4B). The mRNA-seq data also confirmed that global ethylene signaling was induced similarly in old and young leaves during submergence, as indicated by the similar expression of EIN3 target genes between these leaves (Supplemental Figure 4D).

The *ORE1*-dependent response during submergence does not seem to involve ERVII-mediated hypoxia signaling, as none of the 47 out of 51 core hypoxia genes (Mustroph et al., 2009) detected in our dataset differed in expression between Col-0 and *ore1-1* in either old or young leaves (Supplemental Figure 2C). Interestingly, hypoxia represses *ORE1* expression (Supplemental Figure 5A). This result was in accordance with public transcriptome datasets from hypoxia-treated plants, in which expression of *ORE1* and its targets was repressed rather than induced (Supplemental Figure 5B) (Branco-Price et al., 2005; Licausi et al., 2011; Chang et al., 2012; Lee and Bailey-Serres, 2019; Liu et al., 2022). This suggests that *ORE1* expression and downstream target activation were not induced by hypoxia.

Age-dependent ORE1 phosphorylation during submergence is required for downstream target activation

Although ORE1 protein accumulated to higher levels during submergence in young leaves than in old leaves, its downstream targets were activated mostly in old leaves. This indicated an age-dependent activation of ORE1 in old leaves. The transactivation ability of ORE1 was recently shown to depend on its six-fold phosphorylation (Durian et al., 2020). We thus probed this post-translational modification as a potential mechanism mediating differential ORE1 activation in our system. Protein extracts from leaves of submerged *pORE1:ORE1-HA* plants were run on an SDS–PAGE gel containing 50 μM Phos-tag, revealing slower migration of ORE1-HA from old leaves. This suggested the presence of phosphorylated ORE1-HA in old, submerged leaves, supporting our hypothesis of age-dependent ORE1 activation via phosphorylation during submergence (Figure 5A). To further validate this scenario, we used transgenic plants overexpressing a modified ORE1 protein missing the region between amino acids 205 and 221, which contains potential phosphorylation sites (*35S:ORE1Δ17*). These *35S:ORE1Δ17* plants showed a phenotype intermediate between Col-0 and *ore1-1* plants under submergence stress (Figure 5B and 5C).

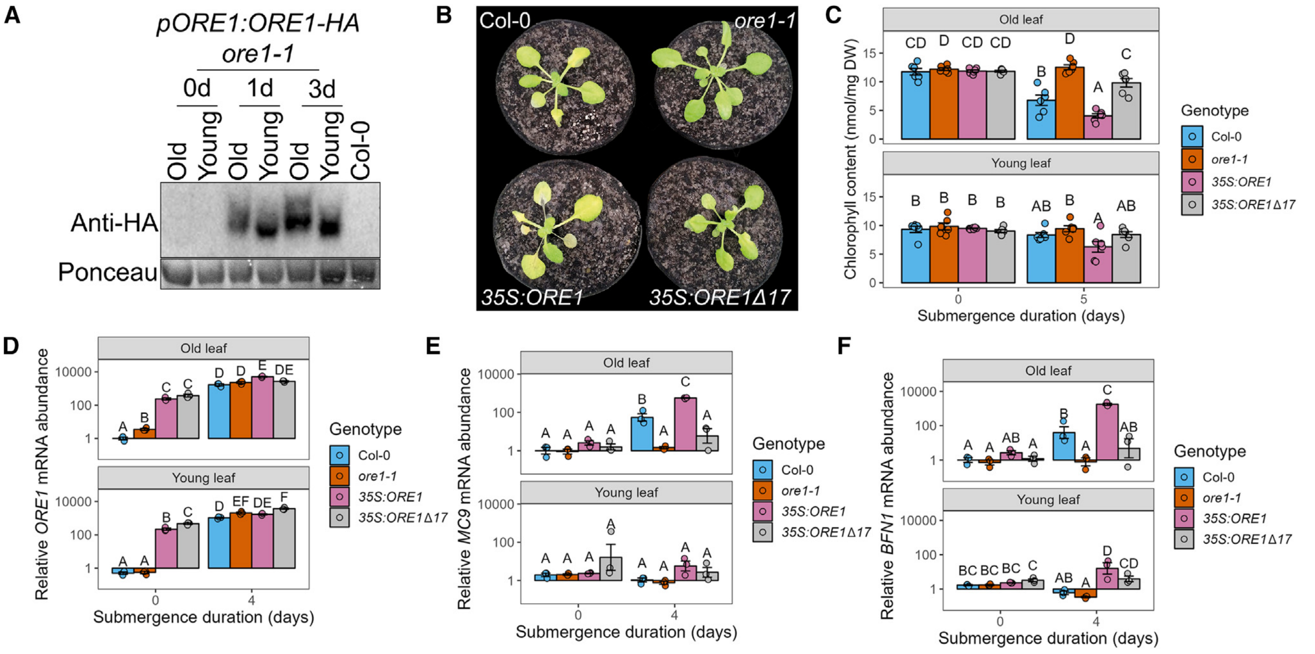

Figure 5. ORE1 phosphorylation during flooding is age dependent

(A) *pORE1:ORE1-HA ore1-1* protein samples from submerged old leaves move more slowly through a Phos-tag gel than samples from young leaves, indicating age-specific phosphorylation of ORE1. Five old or young leaves were pooled together from different plants per *pORE1:ORE1-HA ore1-1* sample, and the Col-0 sample was from one whole rosette.

(B) Representative images of Col-0, *ore1-1*, *35S:ORE1*, and *35S:ORE1Δ17* plants after 5 days of submergence followed by 1 day of recovery.

(C) Chlorophyll content of Col-0, *ore1-1*, *35S:ORE1*, and *35S:ORE1Δ17* plants before and immediately after 5 days of submergence.

(D–F) Expression of *ORE1*, *MC9*, and *BFN1* in Col-0, *ore1-1*, *35S:ORE1*, and *35S:ORE1Δ17* before and after 4 days of submergence. Expression was normalized to that of non-submerged old leaves of Col-0. Two old or young leaves from different plants were pooled together per sample. Different letters indicate significant differences among groups (two-way ANOVA and Tukey's post-hoc test). Error bars indicate SEM.

As expected, expression of *ORE1* was already high before submergence in *35S:ORE1* and *35S:ORE1Δ17* and was also induced in both old and young leaves of Col-0 and *ore1-1* during submergence (Figure 5D). The *ore1-1* mutant is a true null mutant that contains a T-DNA insertion in the last exon. The primer pair used here spans the first intron, explaining the increase in *ORE1* transcript levels in *ore1-1* (Balazadeh et al., 2010; Durian et al., 2020). Although expression of *ORE1* was high during submergence in both old and young leaves of all four genotypes tested here, the downstream target genes *MC9* and *BFN1* were induced only in the old leaves of Col-0 and *35S:ORE1* (Figure 5E and 5F). Taken together, these results suggest that age-dependent phosphorylation of ORE1 is required for activation of its downstream target genes.

Ethylene exposure is sufficient to induce age-dependent accumulation of phosphorylated ORE1

Plants with impaired ethylene signaling did not show age-dependent leaf death during submergence, and treatment with ethylene in darkness induced age-dependent leaf death (Figure 1C–1E). This could not be explained by *ORE1* transcript levels, because ethylene treatment and submergence caused leaf-age-independent *ORE1* induction (Figures 2B and 3B). This also held true for ORE1 protein levels (Figures 3C and 6A). Although the combination of ethylene and darkness was both essential and sufficient for induction of ORE1 protein levels similar to those observed during submergence, this occurred in both old and young leaves (Figure 6A). However, considering that during submergence ORE1 phosphorylation and activation occurred only in old leaves, we hypothesized that ethylene might be the underlying submergence signal (Figure 5A). Consistent with this notion, ethylene exposure in darkness was already sufficient to induce the accumulation of age-dependent phosphorylated ORE1 protein in 1 day (Figure 6B). This result was consistent with the previous observation that ethylene treatment, rather than hypoxia, is sufficient to induce age-dependent leaf yellowing (Figure 1D). Furthermore, treatment with ethylene in darkness had a similar effect on the senescence phenotype of Col-0 plants as submergence in darkness (Figure 6C and 6D). In ethylene-insensitive *ein3eil1* plants, however, this induction of senescence during dark submergence was lost. To probe this effect further, we induced *ORE1* expression throughout the rosette using transgenic plants expressing *ORE1* under an estradiol-responsive promoter. Systemic *ORE1* induction led to age-dependent induction of ORE1 target genes and age-dependent leaf yellowing (supplemental Figure 6). This suggests that flooding-induced ethylene signaling controls systemic ORE1 accumulation but is not essential for age-dependent activation of ORE1. The loss of age-dependent senescence observed in flooded ethylene-insensitive mutants (Figure 1) could likely be an effect of the role of ethylene in leaf development (Vandenbussche et al., 2012).

ORE1 is phosphorylated by CPK1 *in vivo* (Durian et al., 2020). *CPK1* mRNA levels showed a leaf-age-dependent increase

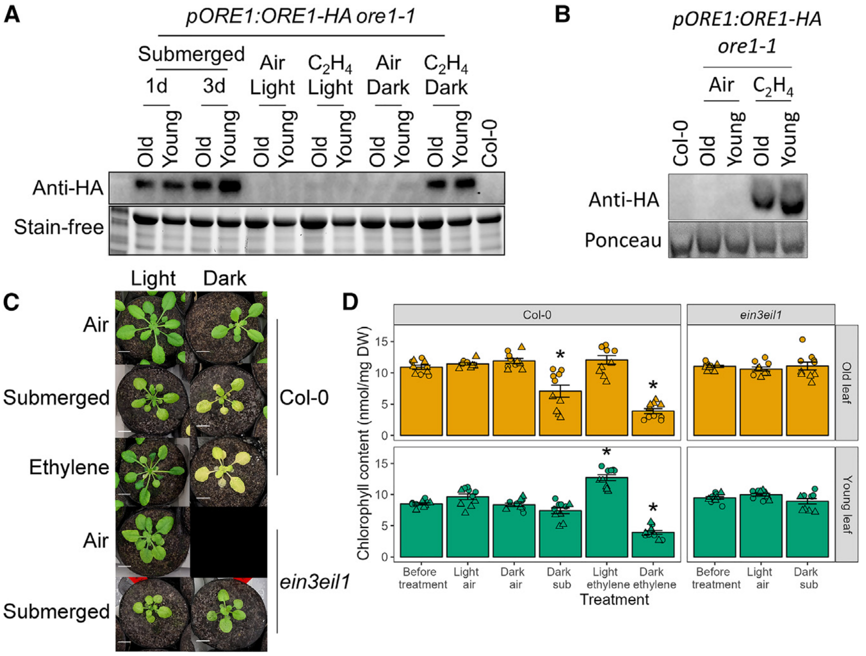

Figure 6. Ethylene controls leaf-age-dependent ORE1 phosphorylation

(A) Immunoblots showing ORE1 accumulation in old and young leaves after submergence or 1 day of ethylene treatment in darkness. Five old or young leaves from different plants were pooled together per *pORE1:ORE1-HA ore1-1* sample. The Col-0 sample was from one whole rosette. Stain-free imaging of the protein gel was used as a loading control.

(B) ORE1-HA from old leaves treated with ethylene in darkness for 1 day moves more slowly through a Phos-tag gel than ORE1-HA from young leaves. Samples are the same as those run on the non-Phos-tag gel in **(A)**. Ponceau staining of the large subunit of Rubisco was used as a loading control.

(C) Shoot phenotypes in response to submergence or ethylene in light or dark conditions. Representative images of Col-0 and *ein3eil1* plants immediately after 5 days of the indicated treatments are shown. Scale bars correspond to 1 cm.

(D) Chlorophyll content of old and young leaves after treatments with different combinations of ethylene and submergence (sub) in light and darkness. Asterisks indicate significant differences from the chlorophyll levels before treatment (one-way ANOVA and Dunnett's test), and error bars indicate SEM. n = 10 per sample from 2 independent experiments; circles and triangles indicate experimental replicates.

in submerged plants, although the absolute changes in expression were small (supplemental Figure 7A). *CPK1* also possesses an EIN3 binding site in its promoter (supplemental Figure 7B). We therefore investigated it as a candidate kinase that might phosphorylate and activate ORE1 downstream of ethylene. However, *CPK1* expression did not change in response to ethylene treatment in either old or young leaves (supplemental Figure 7C). Consistent with this finding, the chlorophyll content of *cpk1-1* mutants did not differ from that of Col-0 after either submergence or ethylene treatment (supplemental Figure 7D–7E). A comparison of Col-0, *ore1-1*, and *cpk1-1* plants revealed age-dependent leaf death in all genotypes upon submergence. This was significantly delayed in *ore1-1* compared with Col-0 but not in *cpk1-1* (supplemental Figure 7F). Thus, although ethylene exposure selectively induces senescence in old leaves via the age-dependent phosphorylation of ORE1, this does not seem to depend on *CPK1*.

Taken together, these results provide a mechanism by which plants ensure that leaf senescence follows an age-dependent gradient during flooding stress. Such a mechanism might safeguard against a total overall collapse of the plant due to high ethylene accumulation during flooding. Interestingly, it is still unclear what prevents ethylene-mediated activation in young leaves. Although flooding stress induces systemic ethylene signaling and ORE1 accumulation, the age-dependent phosphorylation of ORE1 ensures that it can only activate its downstream targets in older tissues (Figure 7). The accumulation of ORE1 in young leaves can prepare them to rapidly transition into senescence if the submergence duration is long enough.

DISCUSSION

Our results demonstrate a mechanism whereby plant responses to a systemic stress cue are determined locally. Submergence of Arabidopsis rosettes activates ethylene signaling in all leaves, consistent with an expected systemic accumulation of ethylene, yet initiates senescence in a specific leaf-age-dependent pattern. Leaf senescence during flooding starts in the oldest leaves but eventually spreads down the age gradient to the younger leaves. Ethylene accumulation and signaling throughout the plant cause this age gradient, whereby the transcription factor ORE1 plays a dominant role in rapidly starting the de-greening process preferentially in older leaves. Although ethylene leads to ORE1 protein accumulation independently of age, ORE1 activation via phosphorylation occurs specifically in the older leaves. Such a mechanism ensures ORE1 target activation and senescence only in these older leaves. Although ORE1 protein was already produced in young leaves within 1 day of submergence, its effects on the transcriptome were minimal during 4 days of submergence. The premature production of ORE1 in young leaves means that, during prolonged submergence, when energy levels are low, senescence can be induced without the need to make new ORE1 protein. In such an instance, ORE1 need only be phosphorylated to induce transcription of its downstream targets.

ORE1 is arguably one of the best-studied transcription factors that control leaf senescence in Arabidopsis. Besides EIN3, other transcription factors also directly induce *ORE1* transcription, including ATAF1, ATAF2, PIF4, PIF5, ABI5, EEL, PRR9, WRKY71, GI, and ARF2 (Sakuraba et al., 2014; Garapati et al., 2015; Kim et al., 2018, 2020; Nagahage et al., 2018; Yu et al., 2021; Xue et al., 2022). *ORE1* mRNA levels are regulated post-transcriptionally by *miR164* (Kim et al., 2009). The low *ORE1* mRNA levels in old leaves under control conditions (Figures 2B and 3B) despite the strong *ORE1* promoter activity (supplemental Figure 3A) suggest that *ORE1* mRNA was rapidly broken down. ORE1 protein levels are controlled by ubiquitination via the E3 ligase NLA and the E2 conjugase PHO2 and by deubiquitination via the ubiquitin-specific proteases UBP12 and UBP13 (Park et al., 2018; 2019).

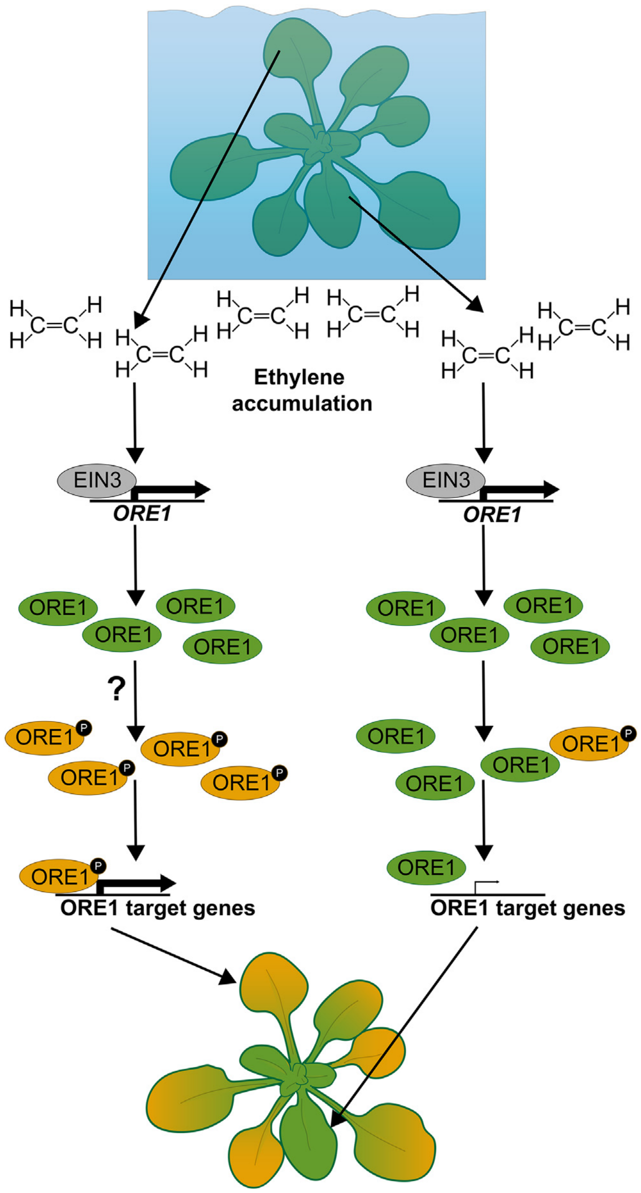

Figure 7. A model for ethylene-mediated sequential leaf senescence in flooded plants

Upon submergence, ethylene rapidly accumulates throughout the plant. This age-independent accumulation of ethylene induces the age-independent accumulation of *ORE1* mRNA and protein via EIN3 stabilization. Leaf-age-dependent senescence is triggered by ethylene via ORE1 phosphorylation and activation specifically in old leaves via an unknown mechanism. This age-dependent phosphorylation of ORE1 ensures that it induces senescence in old leaves; the oldest leaves are thus broken down first and the youngest leaves and meristem last.

Finally, the transactivation activity of ORE1 is activated via phosphorylation by CPK1 (Durian et al., 2020). The established pathway of *miR164*-based inhibition of premature ORE1 accumulation was not sufficient to prevent accumulation of ORE1 in young leaves during flooding. Despite the plethora of regulators that affect the abundance of *ORE1* mRNA and protein, we found that ORE1 abundance did not explain the difference in ORE1 target gene activation between old and young leaves during submergence. Rather, this difference was controlled by post-translational modification of ORE1, which limits its activity to old leaves. ORE1 induces the transcription of its targets via an interaction with the positively charged C terminus of Mediator complex subunit 19a (MED19A), which recruits RNA polymerase II to target genes (Cheng et al., 2022). Phosphorylation of a protein typically reduces its charge, and the phosphorylation of ORE1 could potentially facilitate its binding to MED19a. This is also consistent with the impaired transactivation activity of ORE1Δ17, although it still exhibits DNA binding activity (Durian et al., 2020).

Ethylene can freely diffuse across cell membranes and does not require specific transporters to move between cells. The lack of control of ethylene movement requires a plant to have a highly tissue-specific ethylene response system. This has been described for different cell types (Cao et al., 1999; Polko et al., 2011; Rajhi et al., 2011; Vaseva et al., 2018) and also for similar tissues at different developmental stages (Jing et al., 2005; dela Fuente and Leopold, 1968). Tissue-specific regulation of ethylene responsiveness occurs on many levels of the ethylene signaling cascade (Stepanova and Alonso, 2009). Because ethylene-insensitive mutants do not induce ORE1-mediated senescence of old leaves during flooding, their old leaves die more slowly than those of wild-type plants. The young leaves of ethylene-insensitive mutants, on the other hand, die faster than those of wild-type plants. This could be an effect of the impaired ability of ethylene mutants to respond to reactive oxygen species that accumulate during submergence recovery or of other unidentified roles of ethylene in submergence survival (Tsai et al., 2014; Liu et al., 2022). This highlights how ethylene signaling can lead to either death or survival of a leaf during flooding stress, depending on the age of the leaf.

Our results show that ethylene-induced leaf senescence requires darkness. It is currently unclear whether this is an effect of light signaling or of darkness-induced carbon starvation, as both are known to interact with ethylene signaling (Yanagisawa et al., 2003; Zhong et al., 2012; Shi et al., 2016; Kim et al., 2017). In addition to ethylene accumulation, impaired gas diffusion also leads to a decline in oxygen levels in flooded plants. Hypoxia is also considered an important regulatory signal mediating flood survival responses. Hypoxia by itself does not produce a gradient of age-dependent leaf yellowing, and mutants with impaired hypoxia sensing still show age-dependent leaf death during flooding stress (Figure 1D–1F). Furthermore, core hypoxia genes are not affected by loss of *ORE1*, showing that *ORE1* is not upstream of hypoxia signaling (supplemental Figure 4D). On the basis of these results, we conclude that the sequential leaf death described here does not appear to involve oxygen sensing and signaling mediated by the N-degron pathway. ORE1 and rice SUB1A are both important regulators of the submergence response, but both are controlled primarily by ethylene rather than hypoxia (Gibbs et al., 2011; Lin et al., 2019). This likely stems from the prevalence of hypoxia in normal plant development and the variation in oxygen concentrations among submerged plant tissues (Sasidharan et al., 2018; Weits et al., 2019).

Ethylene accumulation upon submergence induces senescence of old leaves via the age-dependent phosphorylation of ORE1. Our results suggest that this phosphorylation is independent of CPK1, which is known to phosphorylate ORE1 *in vivo* (Durian et al., 2020). Future research should focus on how exactly the

age-dependent phosphorylation of ORE1 is controlled. Protein kinases and phosphatases themselves are often controlled post-translationally, and interactions between them and their targets can be highly context specific (Simeunovic et al., 2016; Bhaskara et al., 2019), potentially complicating the identification of post-translational regulators of ORE1 during submergence.

The severely reduced diffusion of gases in water means that ethylene will accumulate rapidly in any plant tissue that is completely submerged. This property of ethylene makes it an ideal flood warning cue mediating many flood-adaptive traits (Sasidharan and Voesenek, 2015). However, such high concentrations of ethylene mean that senescence is inevitable for submerged leaves. Therefore, a mechanism that prevents the simultaneous indiscriminate breakdown of all leaf tissue in such a situation is essential for prolonging survival. The complex signaling network that mediates submergence-induced senescence underscores the importance of fine spatiotemporal regulation of this process (Bui et al., 2020; Broda et al., 2021; Mishra et al., 2022). During natural plant aging, the genetically coordinated process of chlorophyll breakdown during senescence serves to remobilize nutrients for seed and tuber filling (Yu et al., 2015). The ability to retain chlorophyll has been found to correlate with higher submergence tolerance and improved post-submergence photosynthesis (Alpuerto et al., 2016; Yeung et al., 2018). For example, the submergence-tolerance gene *SUB1A* delays leaf senescence. Like that of *ORE1*, the expression of *SUB1A* is regulated by ethylene (Fukao et al., 2006). Whereas ORE1 induces chlorophyll degradation, *SUB1A* inhibits it during both submergence and darkness and thereby contributes to a quiescence strategy during flooding (Fukao et al., 2006; 2012; Xu et al., 2006). However, as energy reserves become increasingly limited during prolonged submergence, senescence would be a beneficial option. In such a situation, a sequential dismantling of older leaves would make available energy and nutrient reserves that can be redirected to sustain younger leaves and the meristem. This sacrificial use of older leaves would serve to enhance growth and photosynthesis recovery when floodwaters subside. Understanding how plants coordinate which tissues are broken down under stressful conditions could help in developing more stress-tolerant crop varieties, as the role of NAC domain transcription factors in senescence is conserved across many plant species (Podzimska-Sroka et al., 2015).

MATERIALS AND METHODS

Plant material

ore1-1 (SALK_090154): described in He et al. (2005) and ordered from NASC. *ore1-2* (SAIL_694_C04): described in Kim et al. (2020) and ordered from NASC. *cpk1-1* (SALK_096452): described in Durian et al. (2020) and ordered from NASC.

35S:ORE1: described in Matallana-Ramirez et al. (2013); gift from Salma Balazadeh. *35S:ORE1Δ17*: described in Durian et al. (2020); gift from Tina Romeis.

ein2-5: described in Alonso et al. (1999) and ordered from NASC. *ein3eil1*: described in Alonso et al. (2003) and ordered from NASC. *pco124*: described in Masson et al. (2019); gift from Daan Weits.

erfVII: described in Abbas et al. (2015); gift from Daan Weits. *prt6-1*: described in Garzón et al. (2007); gift from Angelika Mustroph. *35S:EIN3-GFP ein3eil1*: described in Xie et al. (2015); gift from Shi Xiao. RPS5aXVE>>ORE1-GFP: described in Gao et al. (2018); gift from Moritz Nowack. *pORE1:ORE1-HA ore1-1*: this study. *pORE1:GUS*: this study.

All Arabidopsis lines were in the ecotype Col-0 (Columbia-0) background.

Generation of transgenic lines

Genomic DNA from a leaf of Arabidopsis ecotype Col-0 was -extracted using phenol:chloroform:isoamyl alcohol. The *ORE1* genomic region, including introns, 5′ UTR, and a 1624-bp promoter, was amplified from this DNA using primers 5383 and 5384 (supplemental Table 2) and inserted into the pJET1.2 vector (Thermo Fisher, K1231) according to the manufacturer's instructions. For the *pORE1:ORE1-HA* line, the entire fragment without the stop codon was amplified from this vector using primers 5383 and 5510, and an HA tag was added using primers 5383 and 5783. For the *pORE1:GUS* line, the *ORE1* promoter was amplified using primers 5383 and 5712. Adapters for binary LIC vectors pPLV01 and pPLV13 (De Rybel et al., 2011) were added to the *pORE1:ORE1-HA* and *pORE1* fragments using primers 5804 and 5761 and 5739 and 5740, respectively. The fragments were inserted into their respective vectors via ligation-independent cloning as described previously (De Rybel et al., 2011). These vectors were introduced into *Agrobacterium tumefaciens* strain AGL-1 via electroporation, and *ore1-1* and Col-0 Arabidopsis plants were transformed using the floral dip method (Logemann et al., 2006). Independent T_1 transformants were selected on plates containing 50 μM Basta/PPT; homozygous T_3 or T_4 lines were used in all experiments.

Plant growth and treatments

Seeds were sown on Primasta soil mix and stratified in the dark for 3–4 days, then transferred to a climate chamber under short-day conditions (20°C, 9-h light, 15-h dark, 70% RH, ∼140–180 PAR either LED or fluorescent light). After germinating for 9 days, seedlings were transplanted to individual pots (5.5 cm diameter, 5 cm height) with a 2:1 perlite:soil mix; pots were covered with a black mesh to prevent soil from floating out during submergence. One liter of 0.5× Hoagland medium was added to each tray of 42 pots. When plants reached the 10-leaf stage, they were submerged in complete darkness at 20°C for the indicated duration and then left to recover for the indicated duration in the original climate chamber. Submergence treatment for the time-lapse videos (supplemental Videos 1 and 2) was performed at 1 PAR, and images were taken every 30 min over 2 weeks using a Nikon D750 camera. Ethylene treatments were performed in 22.5-l desiccators as described in Hartman et al. (2019). Hypoxia treatments were performed by mixing N_2 and air to a concentration of 5% O_2, which was flushed through a desiccator for 1 h. Desiccator valves were then closed, and 5–10 ppm ethylene was injected with a syringe. To quantify leaf death, leaves were scored as dead when more than half of the leaf area

had desiccated after 3 days of post-submergence recovery in the light. Leaves designated as "old" (3–5, Figure 1A) had fully expanded leaf blades, whereas "young" leaves (6–8, Figure 1A) were typically still in the expansion stage at the start of a treatment.

Quantification of green and senescing leaf area

Images of Col-0 and *ore1-1* plants were obtained with a Nokia 8 phone camera. Individual pixels in each image were classified as either "green," "senescing," "dead," or "background" using the Naïve Bayes Multiclass module within PlantCV (Fahlgren et al., 2015). ImageJ was used to count the number of pixels in the green and senescing categories, and this was plotted relative to the number of green pixels before the start of treatment for supplemental Figure 2H. For Figure 2H, the numbers of green and senescing pixels of the same plant were added together and converted into an area in cm^2.

Seed yield

For seed-yield measurements, plants were either kept under short-day control conditions or submerged for 6 days in darkness and then returned to control conditions. Watering was stopped once the first siliques started to dry out, and plants were left to dry out until all siliques had ripened.

Chlorophyll quantification

For chlorophyll measurements, individual old or young leaf blades of the indicated genotypes were cut off and placed into 1.5-ml Eppendorf tubes containing 1 ml DMSO at the indicated time points. Tubes were incubated in a shaking water bath at 60°C for 30 min in darkness and were then left to cool to room temperature (RT) for another 30 min in darkness. A total of 200 µl of each DMSO solution was pipetted into a 96-well plate, and absorption was measured at 647, 664, and 750 nm using a spectrophotometer plate reader (Synergy HT Multi-Detection Microplate Reader; BioTek Instruments). Chlorophyll A was calculated as 13.71 × (664 nm–750 nm) – 2.858 × (647 nm–750 nm), and chlorophyll B was calculated as 22.39 × (647 nm–750 nm) – 5.42 × (664 nm–750 nm). Leaves were dried at 80°C for 48 h before dry-weight measurement on a Mettler-Toledo MX5 microbalance. Total chlorophyll was calculated by adding chlorophyll A and B together and dividing them by the measured dry weight.

Ion leakage

Five leaves per replicate of the indicated tissues were pooled together in a 15-ml tube containing 3 ml distilled water and were gently shaken for 3 h. The concentration of ions in the solution was measured using a Horiba EC-33 conductivity meter. Plant tissue was then boiled for 20 min to destroy all membranes, and ion leakage was measured again to determine the total ion content. Relative ion leakage was calculated as the ratio of the conductivity before boiling to the conductivity after boiling.

Gene expression

RNA was extracted from the indicated tissues using the QIAGEN RNeasy Plant Mini Kit, including an on-column DNAse treatment, according to the manufacturer's instructions. qPCR data shown in Figure 3B and supplemental Figure 3B were obtained using the Spectrum RNA extraction kit (Sigma-Aldrich), followed by DNase treatment using AMPD1 DNase I (Sigma-Aldrich) to ensure that *miR164b* would not be excluded by the size-exclusion limit of the QIAGEN kit.

Extracted RNA was converted into cDNA using RevertAid H Minus Reverse Transcriptase (Thermo Scientific). For qPCR, 20 ng of cDNA was used per 5-µl reaction, using SYBR Green master mix (Bio-Rad) and the primers indicated in supplemental Table 1.

GUS staining

Whole rosettes of 10-leaf *pORE1:GUS* plants were cut off at the indicated time points and fixed in 90% acetone for 20 min. Plants were then washed twice for 10 min in GUS washing buffer (0.1 M phosphate buffer [pH 7], 10 mM EDTA, 2 mM $K_3Fe(CN)_6$) under vacuum and stained with GUS washing solution (0.1 M phosphate buffer [pH 7], 10 mM EDTA, 1 mM $K_3Fe(CN)_6$, 1 mM $K_4Fe(CN)_6 \cdot 3H_2O$, 0.5 mg/ml X-Gluc) for 10 min under vacuum, followed by 20 h at 37°C. Staining was stopped by incubating the plants with 3:1 acetic acid:ethanol for 1 h. The plants were cleaned by washing with 70% ethanol and scanned using an Epson V800 scanner.

RNA sequencing

Between 8 and 16 young and old leaves of Col-0 and *ore1-1* were harvested before submergence, after 4 days of dark submergence, and after 6 h of post-submergence recovery in the light. Additional Col-0 samples were harvested after 2 days (old and young leaves) and 6 days of submergence (young leaves only), and after 1, 3, and 24 h of recovery (old and young leaves). RNA was extracted using the QIAGEN RNeasy Plant Mini Kit. Genomic DNA was removed by treating the samples with AMPD1 DNase I (Sigma-Aldrich). Libraries were constructed by Macrogen using the TruSeq Stranded mRNA LT Sample Prep Kit (Illumina). Libraries were sequenced on an Illumina NovaSeq 6000 platform via paired-end sequencing to obtain 150-bp reads. Sequenced libraries were trimmed of adapter sequences using FastQC (Babraham Bioinformatics). Cleaned reads were aligned to the Araport11 transcriptome using Kallisto (Bray et al., 2016). Genes were identified as differentially expressed when FDR < 0.05 and $|\log_2 FC| > 1$, as calculated using the R packages edgeR and limma (supplemental Table 3). Fold changes and *P* values for all time points were also calculated compared with non-submerged old leaves of Col-0; these were used in supplemental Figures 4D and 7A and can be found in supplemental Table 4.

ORE1 binding site density

To determine the density of ORE1 binding sites in the promoters of putative target genes, genes were selected from the RNA-seq dataset that showed significantly stronger induction in Col-0 old leaves than in *ore1*-1 old leaves after 4 days of dark submergence. Promoters (1-kb upstream and 100-bp downstream of the transcriptional start site) of these 287 genes were extracted from the TAIR9 genome sequence using the GenomicRanges R package (Lawrence et al., 2013). These promoters were scanned for occurrences of the ORE1 motifs $VMGTR_{N5\text{-}6}YACR$ and TDRCGTRHD, allowing one mismatch (Olsen et al., 2005; Matallana-Ramirez et al., 2013). The density of motif centers along the promoter sequences was corrected for the number of scanned promoters and plotted using ggplot2.

Electrophoretic mobility shift assay

EMSAs were performed as described previously (Wu et al., 2012). ORE1-GST protein was purified as described previously (Durian et al., 2020). Binding reactions were performed using the Odyssey infrared EMSA kit (LI-COR) following the manufacturer's instructions. DNA–protein complexes were separated on a 6% (w/v) retardation gel (EC6365BOX, Invitrogen). The DY682 signal was detected using the Odyssey infrared imaging system from LI-COR.

ChIP–qPCR

For ChIP, 10-leaf stage Col-0 and *pORE1:ORE1-HA ore1-1* plants were submerged for 1 day in darkness to induce ORE1-HA protein accumulation. Chromatin was extracted from 1.5 g of whole-rosette tissue for each replicate. Protein–DNA complexes were immunoprecipitated using anti-HA antibodies (Miltenyi Biotec) (Kaufmann et al., 2010). After reversion of the cross-linking, DNA was purified with the QIAquick PCR Purification Kit (QIAGEN) and analyzed by qPCR. Enrichment of ORE1 at the target promoters was calculated relative to Col-0; significance was determined by comparing the enrichment at each of the target loci to that of the negative control (AT2G22180).

Western blotting

Five leaves of the indicated age were pooled together after the indicated treatment and frozen in liquid nitrogen. Protein was extracted using RIPA buffer (Hartman et al., 2019) and quantified using a Pierce BCA kit. Protein (20–50 μg) was loaded onto a stain-free 4%–15% gel (Bio-Rad). The Rubisco large subunit was visualized using stain-free gel imaging. Proteins were transferred from the gel to a 0.2-μm PVDF membrane using a Bio-Rad trans-blot system for 7 min; efficient transfer was verified by imaging the stain-free blot afterward. The blot was blocked overnight at 4°C in TBS-T + 5% milk. Primary antibody (1:1000, anti-GFP [Roche, no. 11814460001] or anti-HA-HRP [Thermo Fisher, 26183-HRP]) was incubated for 1 h at RT. The blot was washed 4 times for 10 min with TBS-T. In the case of anti-GFP blots, the membrane was incubated with a secondary antibody (1:2500 rabbit anti-mouse, Cell Signaling no. 7076) for 1 h at RT, and the membrane was washed 3× with TBS-T and 2× with TBS for 5 min each. The membrane was incubated with Femto (Thermo Fisher) and imaged under a ChemiDoc imaging system (Bio-Rad) to visualize HRP activity.

To identify phosphorylated proteins, 50 μg of protein extract was precipitated by incubating the sample with 4× the volume of the protein sample 100% ice-cold acetone for 1 h at –20°C. After precipitation, the samples were centrifuged for 10 min at 13 000 *g* at 4°C, and the supernatant was removed. The pellet of precipitated proteins was resuspended in 12 μl water and 3 μl 5× sample loading buffer (250 mM Tris [pH 6.8], 25% glycerol, 10% SDS, 0.05% bromophenol blue) containing 5% beta-mercaptoethanol. Samples were boiled for 5 min at 95°C to denature the proteins and were separated via electrophoresis on a SuperSep Phos-Tag 7.5% gel with 50 μM Phos-Tag (198-17981, Fujifilm Wako, Japan). The gel was run at a stable 20 mA for 2.5 h. After electrophoresis, the gel was washed twice in running buffer containing 10 mM EDTA for 10 min and once in running buffer without EDTA. Protein transfer, membrane blocking, antibody incubation, and imaging were performed as described for the non-Phos-tag ORE1-HA western blots. The large subunit of Rubisco was imaged after staining for ORE1-HA using 0.1% (w/v) Ponceau S to verify equal loading.

Estradiol treatment

Entire *RPS5a::XVE>>ORE1-GFP* plants were sprayed twice daily with 100 μM estradiol (from a 20 mM estradiol stock in ethanol) in water or a mock solution (0.5% ethanol). Leaves 1, 3, and 7 were harvested after 8 days and snap-frozen in liquid nitrogen.

Statistical analysis

All statistical tests were performed in R version 3.6.1 as indicated, and differences were deemed significant at $P < 0.05$.

DATA AND CODE AVAILABILITY

RNA-seq data have been deposited at the European Nucleotide Archive under accession number PRJEB57289. Transcript abundance in the RNA-seq data can also be explored in a Shiny app at https://utrecht-university.shinyapps.io/Rankenberg2022/. All other data and code are available from the lead contact upon request.

SUPPLEMENTAL INFORMATION

Supplemental information is available at *Plant Communications Online*.

FUNDING

We would like to thank Bernhard Würzinger and Markus Teige for their input on Phos-tag western blots and Yorrit van de Kaa for harvesting seeds. This work was financially supported by the Netherlands Organization for Scientific Research grant 016.VIDI.171.006 to T.R. and R.S. and grant ALWOP.419 to H.v.V. S.B. thanks the Max Planck Institute of Molecular Plant Physiology (MPIMP) and Leiden University for funding. No conflict of interest declared.

AUTHOR CONTRIBUTIONS

Conceptualization, T.R., H.v.V., and R.S.; investigation, T.R., H.v.V., M.S., C.-Y.L., M.B.D., and E.A.S.; data analysis, T.R., H.v.V., M.S., and E.A.S.; methodology, T.R., H.v.V., M.S., and C.-Y.L.; supervision, S.B. and R.S.; writing – original draft, T.R. and R.S.; writing – review & editing, T.R., H.v.V., S.B., and R.S.; project administration, R.S.; funding acquisition, R.S.

Received: October 27, 2023
Revised: January 19, 2024
Accepted: February 18, 2024
Published: February 19, 2024

REFERENCES

Abbas, M., Berckhan, S., Rooney, D.J., Gibbs, D.J., Vicente Conde, J., Sousa Correia, C., Bassel, G.W., Marín-de la Rosa, N., León, J., Alabadí, D., et al. (2015). Oxygen Sensing Coordinates Photomorphogenesis to Facilitate Seedling Survival. Curr. Biol. **25**:1483–1488.

Alonso, J.M., Hirayama, T., Roman, G., Nourizadeh, S., and Ecker, J.R. (1999). EIN2, a Bifunctional Transducer of Ethylene and Stress Responses in Arabidopsis. Science **284**:2148–2152.

Alonso, J.M., Stepanova, A.N., Solano, R., Wisman, E., Ferrari, S., Ausubel, F.M., and Ecker, J.R. (2003). Five components of the ethylene-response pathway identified in a screen for weak ethylene-insensitive mutants in Arabidopsis. Proc. Natl. Acad. Sci. USA **100**:2992–2997.

Alpuerto, J.B., Hussain, R.M.F., and Fukao, T. (2016). The key regulator of submergence tolerance, SUB1A, promotes photosynthetic and metabolic recovery from submergence damage in rice leaves. Plant Cell Environ. **39**:672–684.

Alvarez, J.M., Brooks, M.D., Swift, J., and Coruzzi, G.M. (2021). Time-Based Systems Biology Approaches to Capture and Model Dynamic Gene Regulatory Networks. Annu. Rev. Plant Biol. **72**:105–131.

Argueso, C.T., Hansen, M., and Kieber, J.J. (2007). Regulation of Ethylene Biosynthesis. J. Plant Growth Regul. **26**:92–105.

Balazadeh, S., Siddiqui, H., Allu, A.D., Matallana-Ramirez, L.P., Caldana, C., Mehrnia, M., Mueller-Roeber, B., Köhler, B., and Mueller-Roeber, B. (2010). A gene regulatory network controlled by the NAC transcription factor ANAC092/AtNAC2/ORE1 during salt-promoted senescence. Plant J. **62**:250–264.

Bhaskara, G.B., Wong, M.M., and Verslues, P.E. (2019). The flip side of phospho-signalling: Regulation of protein dephosphorylation and the protein phosphatase 2Cs. Plant Cell Environ. **42**:2913–2930.

Binder, B.M. (2020). Ethylene signaling in plants. J. Biol. Chem. **295**:7710–7725.

Branco-Price, C., Kawaguchi, R., Ferreira, R.B., and Bailey-Serres, J. (2005). Genome-wide analysis of transcript abundance and translation in Arabidopsis seedlings subjected to oxygen deprivation. Ann. Bot. **96**:647–660.

Bray, N.L., Pimentel, H., Melsted, P., and Pachter, L. (2016). Near-optimal probabilistic RNA-seq quantification. Nat. Biotechnol. **34**:525–527.

Broda, M., Khan, K., O'Leary, B., Pružinská, A., Lee, C.P., Millar, A.H., and Van Aken, O. (2021). Increased expression of ANAC017 primes for accelerated senescence. Plant Physiol. **186**:2205–2221.

Buraschi, F.B., Mollard, F.P.O., Grimoldi, A.A., and Striker, G.G. (2020). Eco-Physiological Traits Related to Recovery from Complete Submergence in the Model Legume Lotus japonicus. Plants **9**:538.

Bui, L.T., Shukla, V., Giorgi, F.M., Trivellini, A., Perata, P., Licausi, F., and Giuntoli, B. (2020). Differential submergence tolerance between juvenile and adult Arabidopsis plants involves the ANAC017 transcription factor. Plant J. **104**:979–994.

Campbell, M.T., Proctor, C.A., Dou, Y., Schmitz, A.J., Phansak, P., Kruger, G.R., Zhang, C., and Walia, H. (2015). Genetic and Molecular Characterization of Submergence Response Identifies Subtol6 as a Major Submergence Tolerance Locus in Maize. PLoS One **10**, e0120385.

Cao, X.F., Linstead, P., Berger, F., Kieber, J., and Dolan, L. (1999). Differential ethylene sensitivity of epidermal cells is involved in the establishment of cell pattern in the Arabidopsis root. Physiol. Plantarum **106**:311–317.

Ceusters, J., and Van de Poel, B. (2018). Ethylene exerts species-specific and age-dependent control of photosynthesis. Plant Physiol. **176**:2601–2612.

Chang, R., Jang, C.J., Branco-Price, C., Nghiem, P., and Bailey-Serres, J. (2012). Transient MPK6 activation in response to oxygen deprivation and reoxygenation is mediated by mitochondria and aids seedling survival in Arabidopsis. Plant Mol. Biol. **78**:109–122.

Chang, K.N., Zhong, S., Weirauch, M.T., Hon, G., Pelizzola, M., Li, H., Huang, S.S.C., Schmitz, R.J., Urich, M.A., Kuo, D., et al. (2013). Temporal transcriptional response to ethylene gas drives growth hormone cross-regulation in Arabidopsis. Elife **2**, e00675.

Chao, Q., Rothenberg, M., Solano, R., Roman, G., Terzaghi, W., and Ecker, J.R. (1997). Activation of the Ethylene Gas Response Pathway in Arabidopsis by the Nuclear Protein ETHYLENE-INSENSITIVE3 and Related Proteins. Cell **89**:1133–1144.

Chen, L., Dodd, I.C., Davies, W.J., and Wilkinson, S. (2013). Ethylene limits abscisic acid- or soil drying-induced stomatal closure in aged wheat leaves. Plant Cell Environ. **36**:1850–1859.

Cheng, S.L.H., Wu, H.-W., Xu, H., Singh, R.M., Yao, T., Jang, I.-C., and Chua, N.-H. (2022). Nutrient status regulates MED19a phase separation for ORESARA1-dependent senescence. New Phytol. **236**:1779–1795. https://doi.org/10.1111/nph.18478.

De Rybel, B., van den Berg, W., Lokerse, A., Liao, C.-Y., van Mourik, H., Möller, B., Peris, C.L., and Weijers, D. (2011). A versatile set of ligation-independent cloning vectors for functional studies in plants. Plant Physiol. **156**:1292–1299.

Doubt, S.L. (1917). The Response of Plants to Illuminating Gas. Bot. Gaz. **63**:209–224.

Dubois, M., Van den Broeck, L., and Inzé, D. (2018). The Pivotal Role of Ethylene in Plant Growth. Trends Plant Sci. **23**:311–323.

Durian, G., Sedaghatmehr, M., Matallana-Ramirez, L.P., Schilling, S.M., Schaepe, S., Guerra, T., Herde, M., Witte, C.-P., Mueller-Roeber, B., Schulze, W.X., et al. (2020). Calcium-Dependent Protein Kinase CPK1 Controls Cell Death by In Vivo Phosphorylation of Senescence Master Regulator ORE1. Plant Cell **32**:1610–1625.

Fahlgren, N., Feldman, M., Gehan, M.A., Wilson, M.S., Shyu, C., Bryant, D.W., Hill, S.T., McEntee, C.J., Warnasooriya, S.N., Kumar, I., et al. (2015). A Versatile Phenotyping System and Analytics Platform Reveals Diverse Temporal Responses to Water Availability in Setaria. Mol. Plant **8**:1520–1535.

dela Fuente, R.K., and Leopold, A.C. (1968). Senescence Processes in Leaf Abscission. Plant Physiol. **43**:1496–1502.

Fukao, T., and Bailey-Serres, J. (2008). Submergence tolerance conferred by Sub1A is mediated by SLR1 and SLRL1 restriction of gibberellin responses in rice. Proc. Natl. Acad. Sci. USA **105**:16814–16819.

Fukao, T., Xu, K., Ronald, P.C., and Bailey-Serres, J. (2006). A Variable Cluster of Ethylene Response Factor–Like Genes Regulates Metabolic and Developmental Acclimation Responses to Submergence in Rice. Plant Cell **18**:2021–2034.

Fukao, T., Yeung, E., and Bailey-Serres, J. (2012). The Submergence Tolerance Gene SUB1A Delays Leaf Senescence under Prolonged Darkness through Hormonal Regulation in Rice. Plant Physiol. **160**:1795–1807.

Gao, Z., Daneva, A., Salanenka, Y., Van Durme, M., Huysmans, M., Lin, Z., De Winter, F., Vanneste, S., Karimi, M., Van de Velde, J., et al. (2018). KIRA1 and ORESARA1 terminate flower receptivity by promoting cell death in the stigma of Arabidopsis. Nat. Plants **4**:365–375.

Garapati, P., Feil, R., Lunn, J.E., Van Dijck, P., Balazadeh, S., and Mueller-Roeber, B. (2015). Transcription Factor Arabidopsis Activating Factor1 Integrates Carbon Starvation Responses with Trehalose Metabolism. Plant Physiol. **169**:379–390.

Garzón, M., Eifler, K., Faust, A., Scheel, H., Hofmann, K., Koncz, C., Yephremov, A., and Bachmair, A. (2007). PRT6/At5g02310 encodes an Arabidopsis ubiquitin ligase of the N-end rule pathway with arginine specificity and is not the CER3 locus. FEBS Lett. **581**:3189–3196.

Gibbs, D.J., Lee, S.C., Isa, N.M., Gramuglia, S., Fukao, T., Bassel, G.W., Correia, C.S., Corbineau, F., Theodoulou, F.L., Bailey-Serres, J., et al. (2011). Homeostatic response to hypoxia is regulated by the N-end rule pathway in plants. Nature **479**:415–418.

Graham, L.E., Schippers, J.H.M., Dijkwel, P.P., and Wagstaff, C. (2012). Ethylene and senescence processes. Annual Plant Reviews **44**:305–341.

Hartman, S., Liu, Z., van Veen, H., Vicente, J., Reinen, E., Martopawiro, S., Zhang, H., van Dongen, N., Bosman, F., Bassel, G.W., et al.

(2019). Ethylene-mediated nitric oxide depletion pre-adapts plants to hypoxia stress. Nat. Commun. **10**:4020.

Hattori, Y., Nagai, K., Furukawa, S., Song, X.J., Kawano, R., Sakakibara, H., Wu, J., Matsumoto, T., Yoshimura, A., Kitano, H., et al. (2009). The ethylene response factors SNORKEL1 and SNORKEL2 allow rice to adapt to deep water. Nature **460**:1026–1030.

Havé, M., Marmagne, A., Chardon, F., and Masclaux-Daubresse, C. (2017). Nitrogen remobilization during leaf senescence: lessons from Arabidopsis to crops. J. Exp. Bot. **68**:2513–2529.

He, X.-J., Mu, R.-L., Cao, W.-H., Zhang, Z.-G., Zhang, J.-S., and Chen, S.-Y. (2005). AtNAC2, a transcription factor downstream of ethylene and auxin signaling pathways, is involved in salt stress response and lateral root development. Plant J. **44**:903–916.

Jing, H.-C., Schippers, J.H.M., Hille, J., and Dijkwel, P.P. (2005). Ethylene-induced leaf senescence depends on age-related changes and OLD genes in Arabidopsis. J. Exp. Bot. **56**:2915–2923.

Kaufmann, K., Muiño, J.M., Østerås, M., Farinelli, L., Krajewski, P., and Angenent, G.C. (2010). Chromatin immunoprecipitation (ChIP) of plant transcription factors followed by sequencing (ChIP-SEQ) or hybridization to whole genome arrays (ChIP-CHIP). Nat. Protoc. **5**:457–472.

Kim, C., Kim, S.J., Jeong, J., Park, E., Oh, E., Park, Y.-I., Lim, P.O., and Choi, G. (2020). High Ambient Temperature Accelerates Leaf Senescence via PHYTOCHROME-INTERACTING FACTOR 4 and 5 in Arabidopsis. Mol. Cell. **43**:645–661.

Kim, G.-D., Cho, Y.-H., and Yoo, S.-D. (2017). Regulatory Functions of Cellular Energy Sensor SNF1-Related Kinase1 for Leaf Senescence Delay through ETHYLENE- INSENSITIVE3 Repression. Sci. Rep. **7**:3193.

Kim, H.J., Park, J.H., Kim, J., Kim, J.J., Hong, S., Kim, J., Kim, J.H., Woo, H.R., Hyeon, C., Lim, P.O., et al. (2018). Time-evolving genetic networks reveal a NAC troika that negatively regulates leaf senescence in *Arabidopsis*. Proc. Natl. Acad. Sci. USA **115**:4930–4939.

Kim, J.H., Woo, H.R., Kim, J., Lim, P.O., Lee, I.C., Choi, S.H., Hwang, D., and Nam, H.G. (2009). Trifurcate feed-forward regulation of age-dependent cell death involving *miR164* in *Arabidopsis*. Science **323**:1053–1057.

Krishnan, P., Ravi, I., and Rama Krishnayya, G. (1999). Leaf Senescence in Submerged Rice Plants. Exp. Agric. **35**:345–355.

Kuroha, T., Nagai, K., Gamuyao, R., Wang, D.R., Furuta, T., Nakamori, M., Kitaoka, T., Adachi, K., Minami, A., Mori, Y., et al. (2018). Ethylene-gibberellin signaling underlies adaptation of rice to periodic flooding. Science **361**:181–186.

Lawrence, M., Huber, W., Pagès, H., Aboyoun, P., Carlson, M., Gentleman, R., Morgan, M.T., and Carey, V.J. (2013). Software for Computing and Annotating Genomic Ranges. PLoS Comput. Biol. **9**, e1003118.

Lee, T.A., and Bailey-Serres, J. (2019). Integrative analysis from the epigenome to translatome uncovers patterns of dominant nuclear regulation during transient stress. Plant Cell **31**:2573–2595.

Leeggangers, H.A., Rodriguez-Granados, N.Y., Macias-Honti, M.G., and Sasidharan, R. (2023). A helping hand when drowning: the versatile role of ethylene in root flooding resilience. Environ. Exp. Bot. **213**, 105422.

Li, Z., Peng, J., Wen, X., and Guo, H. (2013). *ETHYLENE-INSENSITIVE3* is a senescence-associated gene that accelerates age-dependent leaf senescence by directly repressing *miR164* transcription in *Arabidopsis*. Plant Cell **25**:3311–3328.

Licausi, F., Kosmacz, M., Weits, D.A., et al. (2011). Oxygen sensing in plants is mediated by an N-end rule pathway for protein destabilization. Nature **479**:419–422.

Lin, C.-C., Chao, Y.T., Chen, W.C., Ho, H.Y., Chou, M.Y., Li, Y.R., Wu, Y.L., Yang, H.A., Hsieh, H., Lin, C.S., et al. (2019). Regulatory cascade involving transcriptional and N-end rule pathways in rice under submergence. Proc. Natl. Acad. Sci. USA **116**:3300–3309.

Liu, Z., Hartman, S., van Veen, H., Zhang, H., Leeggangers, H.A.C.F., Martopawiro, S., Bosman, F., de Deugd, F., Su, P., Hummel, M., et al. (2022). Ethylene augments root hypoxia tolerance via growth cessation and reactive oxygen species amelioration. Plant Physiol. **190**:1365–1383.

Logemann, E., Birkenbihl, R.P., Ülker, B., and Somssich, I.E. (2006). An improved method for preparing Agrobacterium cells that simplifies the Arabidopsis transformation protocol. Plant Methods **2**:16.

Masson, N., Keeley, T.P., Giuntoli, B., White, M.D., Puerta, M.L., Perata, P., Hopkinson, R.J., Flashman, E., Licausi, F., and Ratcliffe, P.J. (2019). Conserved N-terminal cysteine dioxygenases transduce responses to hypoxia in animals and plants. Science **365**:65–69.

Matallana-Ramirez, L.P., Rauf, M., Farage-Barhom, S., Dortay, H., Xue, G.-P., Dröge-Laser, W., Lers, A., Balazadeh, S., and Mueller-Roeber, B. (2013). NAC transcription factor ORE1 and senescence-induced BIFUNCTIONAL NUCLEASE1 (BFN1) constitute a regulatory cascade in Arabidopsis. Mol. Plant **6**:1438–1452.

Métraux, J.P., and Kende, H. (1983). The Role of Ethylene in the Growth Response of Submerged Deep Water Rice. Plant Physiol. **72**:441–446.

Mishra, V., Singh, A., Gandhi, N., Sarkar Das, S., Yadav, S., Kumar, A., and Sarkar, A.K. (2022). A unique miR775-GALT9 module regulates leaf senescence in Arabidopsis during post-submergence recovery by modulating ethylene and the abscisic acid pathway. Development **149**:dev199974.

Mustroph, A., Zanetti, M.E., Jang, C.J.H., Holtan, H.E., Repetti, P.P., Galbraith, D.W., Girke, T., and Bailey-Serres, J. (2009). Profiling translatomes of discrete cell populations resolves altered cellular priorities during hypoxia in Arabidopsis. Proc. Natl. Acad. Sci. USA **106**:18843–18848.

Nagahage, I.S.P., Sakamoto, S., Nagano, M., Ishikawa, T., Kawai-Yamada, M., Mitsuda, N., and Yamaguchi, M. (2018). An NAC domain transcription factor ATAF2 acts as transcriptional activator or repressor dependent on promoter context. Plant Biotechnol. **35**:285–289.

Olsen, A.N., Ernst, H.A., Leggio, L.L., and Skriver, K. (2005). DNA-binding specificity and molecular functions of NAC transcription factors. Plant Sci. **169**:785–797.

Park, B.S., Yao, T., Seo, J.S., Wong, E.C.C., Mitsuda, N., Huang, C.-H., and Chua, N.-H. (2018). Arabidopsis NITROGEN LIMITATION ADAPTATION regulates ORE1 homeostasis during senescence induced by nitrogen deficiency. Nat. Plants **4**:898–903.

Park, S.-H., Jeong, J.S., Seo, J.S., Park, B.S., and Chua, N.-H. (2019). Arabidopsis ubiquitin-specific proteases UBP12 and UBP13 shape ORE1 levels during leaf senescence induced by nitrogen deficiency. New Phytol. **223**:1447–1460.

Peng, H.-P., Chan, C.-S., Shih, M.-C., and Yang, S.F. (2001). Signaling Events in the Hypoxic Induction of Alcohol Dehydrogenase Gene in Arabidopsis. Plant Physiol. **126**:742–749.

Podzimska-Sroka, D., O'Shea, C., Gregersen, P.L., and Skriver, K. (2015). NAC Transcription Factors in Senescence: From Molecular Structure to Function in Crops. Plants **4**:412–448.

Polko, J.K., Voesenek, L.A.C.J., Peeters, A.J.M., and Pierik, R. (2011). Petiole hyponasty: an ethylene-driven, adaptive response to changes in the environment. AoB PLANTS **2011**:plr031.

Qiu, K., Li, Z., Yang, Z., Chen, J., Wu, S., Zhu, X., Gao, S., Gao, J., Ren, G., Kuai, B., et al. (2015). EIN3 and ORE1 Accelerate Degreening during Ethylene-Mediated Leaf Senescence by Directly Activating Chlorophyll Catabolic Genes in Arabidopsis. PLoS Genet. **11**, e1005399.

Rajhi, I., Yamauchi, T., Takahashi, H., Nishiuchi, S., Shiono, K., Watanabe, R., Mliki, A., Nagamura, Y., Tsutsumi, N., Nishizawa, N.K., et al. (2011). Identification of genes expressed in maize root cortical cells during lysigenous aerenchyma formation using laser microdissection and microarray analyses. New Phytol. **190**:351–368.

Rankenberg, T., Geldhof, B., van Veen, H., Holsteens, K., Van de Poel, B., and Sasidharan, R. (2021). Age-Dependent Abiotic Stress Resilience in Plants. Trends Plant Sci. **26**:692–705.

Rauf, M., Arif, M., Fisahn, J., Xue, G.-P., Balazadeh, S., and Mueller-Roeber, B. (2013). NAC Transcription Factor SPEEDY HYPONASTIC GROWTH Regulates Flooding-Induced Leaf Movement in Arabidopsis. Plant Cell **25**:4941–4955.

Sakuraba, Y., Jeong, J., Kang, M.Y., Kim, J., Paek, N.C., and Choi, G. (2014). Phytochrome-interacting transcription factors PIF4 and PIF5 induce leaf senescence in Arabidopsis. Nat. Commun. **5**:4636.

Sasidharan, R., Hartman, S., Liu, Z., Martopawiro, S., Sajeev, N., van Veen, H., Yeung, E., and Voesenek, L.A.C.J. (2018). Signal dynamics and interactions during flooding stress. Plant Physiol. **176**:1106–1117.

Sasidharan, R., and Voesenek, L.A.C.J. (2015). Ethylene-Mediated Acclimations to Flooding Stress. Plant Physiol. **169**:3–12.

Shi, H., Shen, X., Liu, R., Xue, C., Wei, N., Deng, X.W., and Zhong, S. (2016). The Red Light Receptor Phytochrome B Directly Enhances Substrate-E3 Ligase Interactions to Attenuate Ethylene Responses. Dev. Cell **39**:597–610.

Simeunovic, A., Mair, A., Wurzinger, B., and Teige, M. (2016). Know where your clients are: subcellular localization and targets of calcium-dependent protein kinases. J. Exp. Bot. **67**:3855–3872.

Stepanova, A.N., and Alonso, J.M. (2009). Ethylene signaling and response: where different regulatory modules meet. Curr. Opin. Plant Biol. **12**:548–555.

Tsai, K.-J., Chou, S.-J., and Shih, M.-C. (2014). Ethylene plays an essential role in the recovery of Arabidopsis during post-anaerobiosis reoxygenation. Plant Cell Environ. **37**:2391–2405.

Vandenbussche, F., Vaseva, I., Vissenberg, K., and Van Der Straeten, D. (2012). Ethylene in vegetative development: a tale with a riddle. New Phytol. **194**:895–909.

Vaseva, I.I., Qudeimat, E., Potuschak, T., Du, Y., Genschik, P., Vandenbussche, F., and Van Der Straeten, D. (2018). The plant hormone ethylene restricts Arabidopsis growth via the epidermis. Proc. Natl. Acad. Sci. USA **115**:E4130–E4139.

Vashisht, D., Hesselink, A., Pierik, R., Ammerlaan, J.M.H., Bailey-Serres, J., Visser, E.J.W., Pedersen, O., van Zanten, M., Vreugdenhil, D., Jamar, D.C.L., et al. (2011). Natural variation of submergence tolerance among Arabidopsis thaliana accessions. New Phytol. **190**:299–310.

Voesenek, L.A.C.J., and Sasidharan, R. (2013). Ethylene – and oxygen signalling – drive plant survival during flooding. Plant Biol. **15**:426–435.

Weits, D.A., Kunkowska, A.B., Kamps, N.C.W., Portz, K.M.S., Packbier, N.K., Nemec Venza, Z., Gaillochet, C., Lohmann, J.U., Pedersen, O., van Dongen, J.T., et al. (2019). An apical hypoxic niche sets the pace of shoot meristem activity. Nature **569**:714–717.

Woo, H.R., Kim, H.J., Lim, P.O., and Nam, H.G. (2019). Leaf senescence: Systems and dynamics aspects. Annu. Rev. Plant Biol. **70**:347–376.

Wu, A., Allu, A.D., Garapati, P., Siddiqui, H., Dortay, H., Zanor, M.I., Asensi-Fabado, M.A., Munné-Bosch, S., Antonio, C., Tohge, T., et al. (2012). *JUNGBRUNNEN1*, a reactive oxygen species–responsive NAC transcription factor, regulates longevity in *Arabidopsis*. Plant Cell **24**:482–506.

Xie, L.-J., Chen, Q.F., Chen, M.X., Yu, L.J., Huang, L., Chen, L., Wang, F.Z., Xia, F.N., Zhu, T.R., Wu, J.X., et al. (2015). Unsaturation of Very-Long-Chain Ceramides Protects Plant from Hypoxia-Induced Damages by Modulating Ethylene Signaling in Arabidopsis. PLoS Genet. **11**, e1005143.

Xu, K., Xu, X., Fukao, T., Canlas, P., Maghirang-Rodriguez, R., Heuer, S., Ismail, A.M., Bailey-Serres, J., Ronald, P.C., and Mackill, D.J. (2006). Sub1A is an ethylene-response-factor-like gene that confers submergence tolerance to rice. Nature **442**:705–708.

Xue, H., Meng, J., Lei, P., Cao, Y., An, X., Jia, M., Li, Y., Liu, H., Sheen, J., Liu, X., et al. (2022). ARF2-PIF5 interaction controls transcriptional reprogramming in the ABS3-mediated plant senescence pathway. EMBO J. **41**, e110988.

Yanagisawa, S., Yoo, S.-D., and Sheen, J. (2003). Differential regulation of EIN3 stability by glucose and ethylene signalling in plants. Nature **425**:521–525.

Yeung, E., van Veen, H., Vashisht, D., Sobral Paiva, A.L., Hummel, M., Rankenberg, T., Steffens, B., Steffen-Heins, A., Sauter, M., de Vries, M., et al. (2018). A stress recovery signaling network for enhanced flooding tolerance in Arabidopsis thaliana. Proc. Natl. Acad. Sci. USA **115**:E6085–E6094.

Yu, S.M., Lo, S.F., and Ho, T.H. (2015). Source–sink communication: regulated by hormone, nutrient, and stress cross-signaling. Trends Plant Sci. **20**:844–857.

Yu, Y., Qi, Y., Xu, J., Dai, X., Chen, J., Dong, C.-H., and Xiang, F. (2021). Arabidopsis WRKY71 regulates ethylene-mediated leaf senescence by directly activating EIN2, ORE1 and ACS2 genes. Plant J. **107**:1819–1836.

Zhang, Y., Gao, Y., Wang, H.-L., Kan, C., Li, Z., Yang, X., Yin, W., Xia, X., Nam, H.G., Li, Z., et al. (2021). Verticillium dahliae secretory effector PevD1 induces leaf senescence by promoting ORE1-mediated ethylene biosynthesis. Mol. Plant **14**:1901–1917.

Zhong, S., Shi, H., Xue, C., Wang, L., Xi, Y., Li, J., Quail, P.H., Deng, X.W., and Guo, H. (2012). A Molecular Framework of Light-Controlled Phytohormone Action in Arabidopsis. Curr. Biol. **22**:1530–1535.

Zhang, J., Toai, T.V., Huynh, L., and Preiszner, J. (1997). Development of Flooding-Tolerant Arabidopsis thaliana by Autoregulated Cytokinin Production, p. 10.

Plant Communications, Volume *5*

Supplemental information

Differential leaf flooding resilience in *Arabidopsis thaliana* is controlled by ethylene signaling-activated and age-dependent phosphorylation of ORESARA1

Tom Rankenberg, Hans van Veen, Mastoureh Sedaghatmehr, Che-Yang Liao, Muthanna Biddanda Devaiah, Evelien A. Stouten, Salma Balazadeh, and Rashmi Sasidharan

Differential leaf flooding resilience in *Arabidopsis thaliana* is controlled by ethylene signaling-activated and age-dependent phosphorylation of ORESARA1 activity

Tom Rankenberg[1], Hans van Veen[1,2], Mastoureh Sedaghatmehr[3], Che-Yang Liao[4], Muthanna Biddanda Devaiah[4], Evelien A Stouten[1], Salma Balazadeh[5], Rashmi Sasidharan[1]

SUPPLEMENTARY INFORMATION

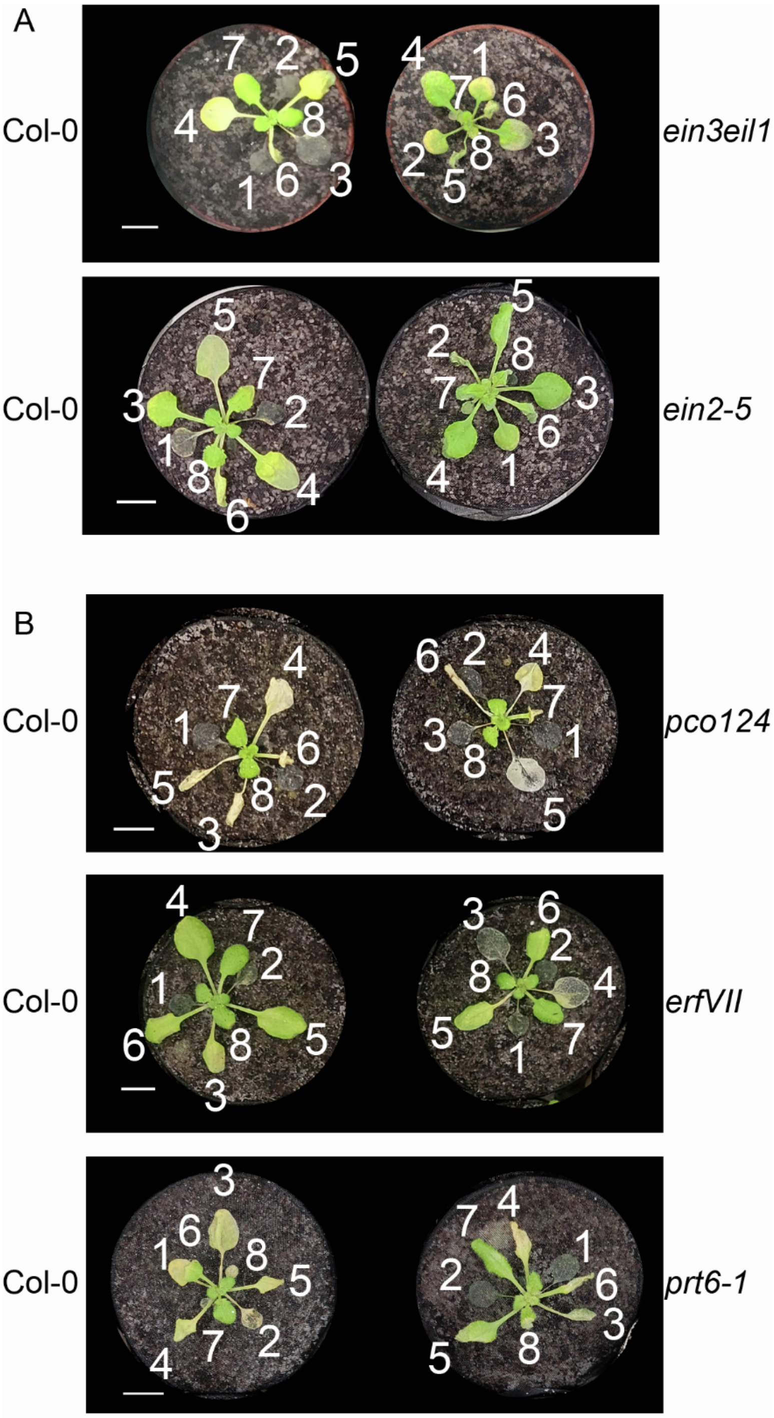

Figure S1

A) Representative images of *ein3eil1* and *ein2-5* mutants after 5 (*ein2-5*/Col-0) or 8 (*ein3eil1*/Col-0) days of submergence followed by one day of recovery

B) Representative images of *pco124*, *erfVII*, and *prt6-1* mutants after 5 (Col-0/*pco124*) or 4 (Col-0/*erfVII*, Col-0/*prt6-1*) days of submergence followed by one day of recovery.

Numbers indicate leaf numbers based on the order of emergence. Scale bars indicate 1cm

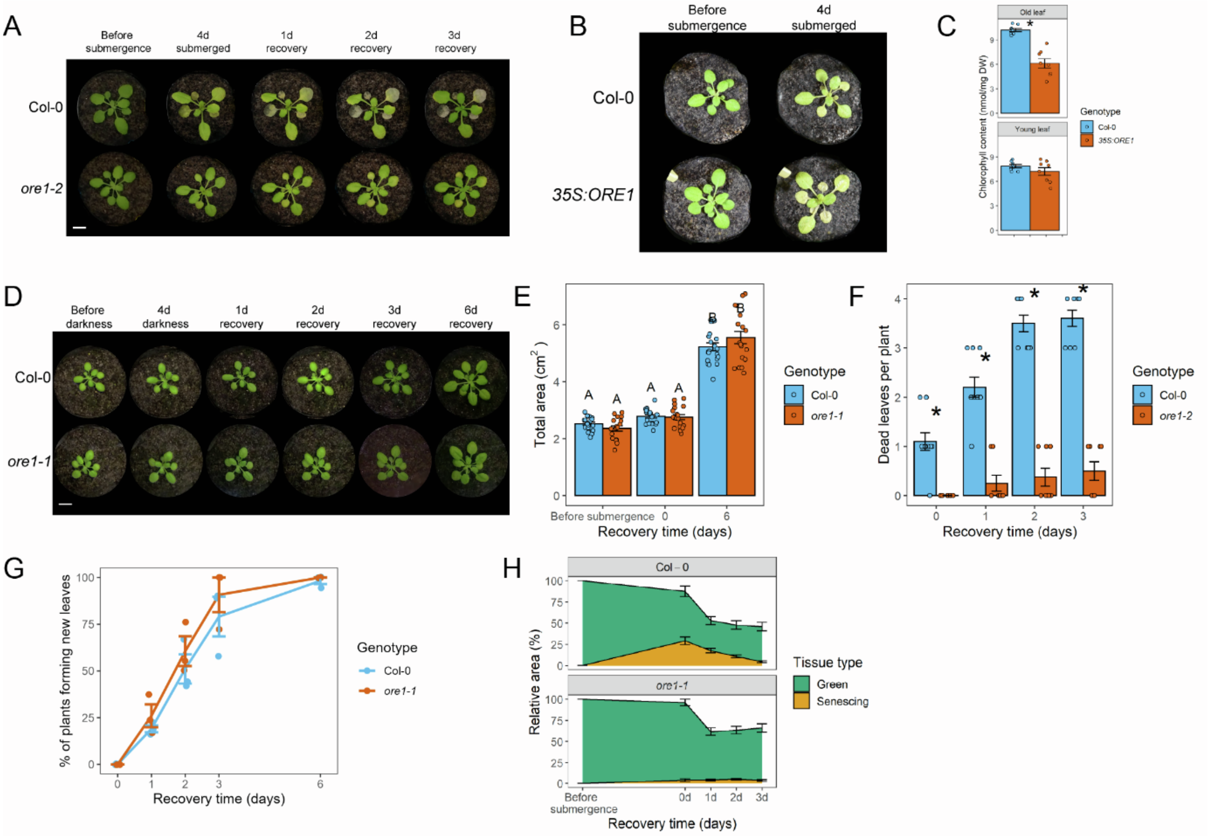

Figure S2

A) *ore1-2* mutants show reduced yellowing of old leaves after recovery from four days of submergence. Representative images of Col-0 and *ore1-2* plants at the indicated timepoints.
B) *35S:ORE1* plants show increased leaf senescence after 4 days of submergence. Representative images of Col-0 and *35S:ORE1* plants at the indicated timepoints.
C) Chlorophyll content of old and young leaves of Col-0 and *35S:ORE1* plants after 4 days of submergence. Asterisks indicate signficant differences (t-test)
D) Four days of darkness does not induce leaf senescence in Col-0 or *ore1-1*. Representative images of Col-0 and *ore1-1* plants at the indicated timepoints.
E) Four days of darkness does not affect the rosette area of Arabidopsis plants in an *ORE1*-dependent manner. Different letters indicate significant differences between groups (two-way ANOVA + Tukey's post-hoc test). n=16-20 per genotype.
F) Dead leaves of Col-0 and *ore1-2* during recovery from 5 days of submergence. Asterisks indicate signficant differences (t-test), n=8-10.
G) The rate of new leaf formation while recovering from 5 days of submergence is not significantly different between Col-0 and *ore1-1* plants (t-test). Dots represent three independent experiments, consisting of 8-21 plants per genotype each.
H) Relative green and senescing areas of Col-0 and *ore1-1* before and after 4 days of submergence, and at the indicated recovery timepoints, as quantified by PlantCV. n =17 (Col-0), 20 (*ore1-1*).
Scale bars indicate 1cm

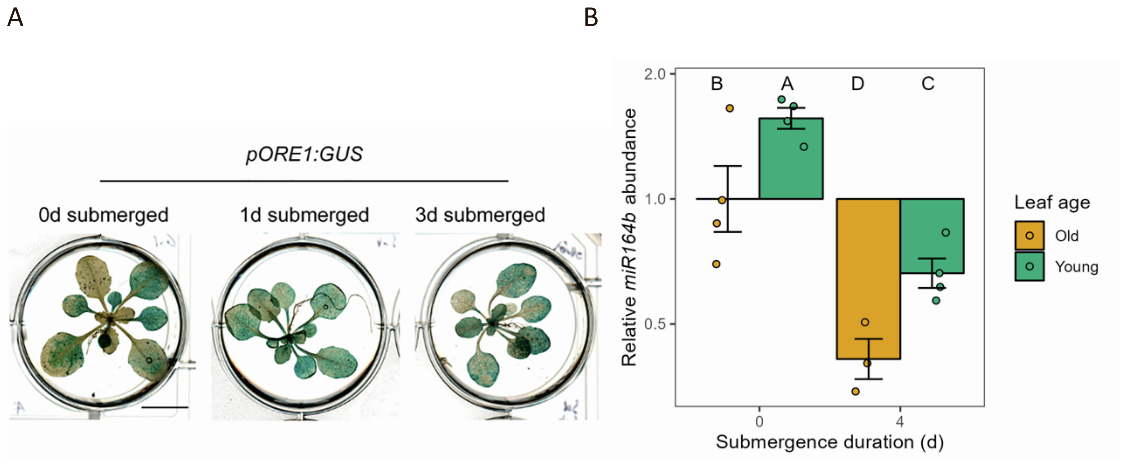

Figure S3

A) GUS staining of *pORE1:GUS* shows that promoter activity is limited to old leaves and cotyledons in non-submerged plants but is induced in all leaves upon submergence. Scale bar indicates 1 cm.

B) *miR164b* pri-miRNA abundance in old and young leaves before and after four days of submergence. Different letters indicate significant differences between groups (two-way ANOVA + Tukey's post-hoc test). n=4 biological replicates for both control and submerged plants. Each replicate consisted of old or young leaves (two per plant) pooled from two plants.

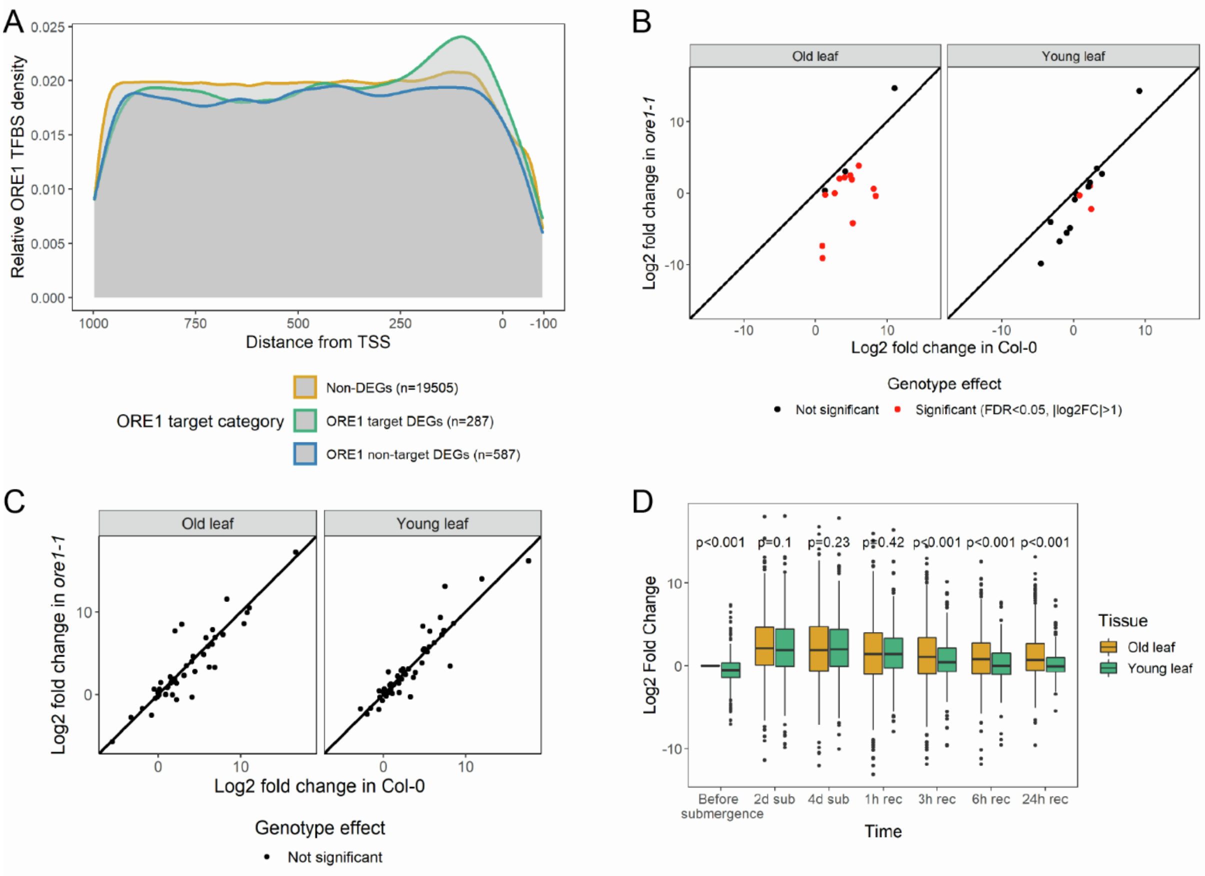

Figure S4
A) Density of *ORE1* binding sites (TDRCGTRHD/VMGTRN5-6YACR, Olsen et al., 2005; Matallana-Ramirez et al., 2013) in the promoters of Non-DEGs, DEGs with lower expression in *ore1-1* than Col-0 in old leaves (ORE1 target DEGs), and all other DEGs between Col-0 and *ore1-1* in old leaves.
B) *In vivo* confirmed ORE1 target genes (n=15, supplemental table S3) show more differences between Col-0 and *ore1-1* in their response to four days of submergence in old leaves than in young leaves.
C) The response of none of the core hypoxia genes (Mustroph et al., 2009) to four days of submergence is significantly different between Col-0 and *ore1-1*.
D) Expression of EIN3 target genes (Chang et al., 2013) is similar in old and young leaves during submergence but is higher in old leaves before and after submergence (Wilcoxon Rank Sum test).

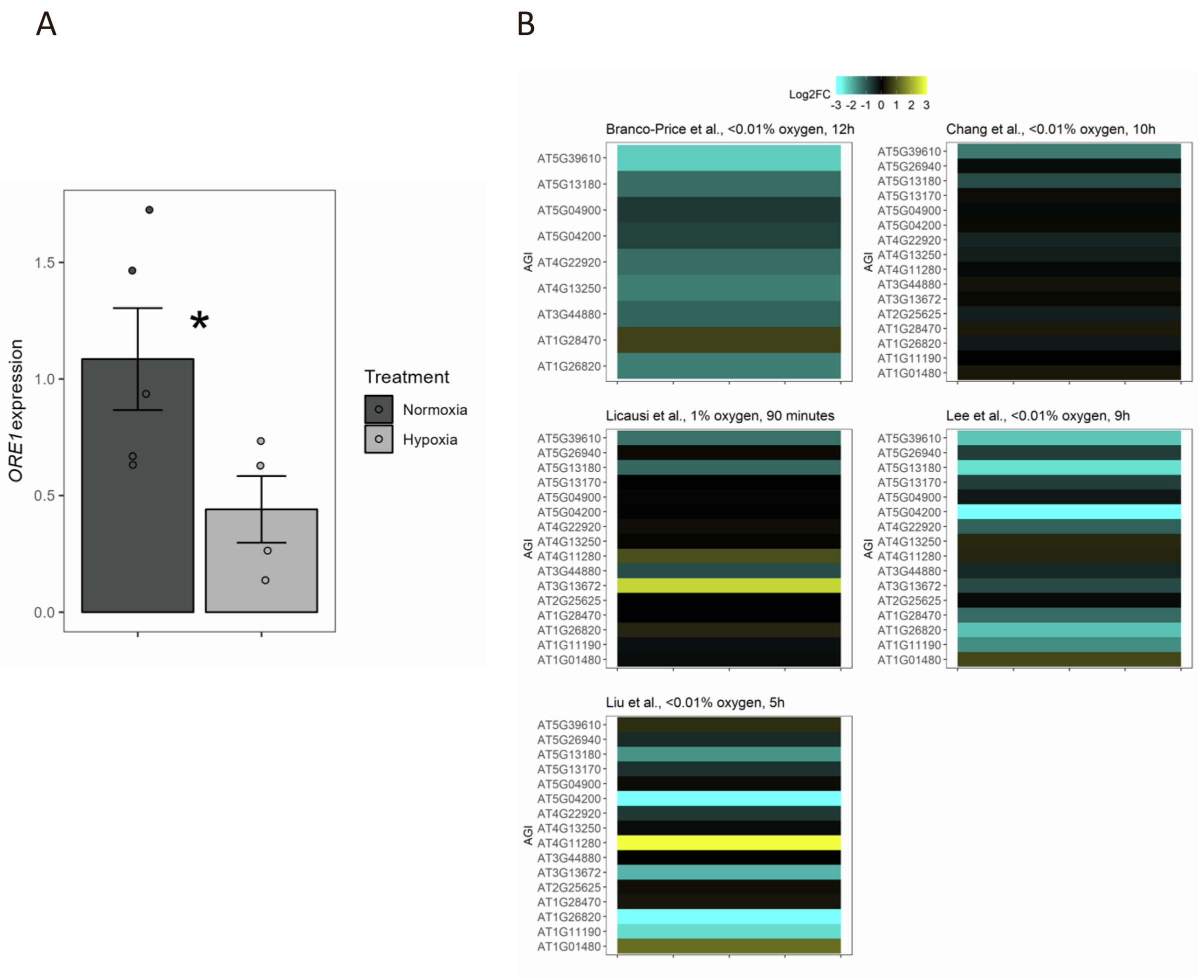

Figure S5

A) The expression of *ORE1* in response to 4h of hypoxia (Col-0 plants), calculated relative to controls in air (normoxia). n=5 for normoxia and n=4 for hypoxia (one rosette per replicate) Asterisks denote a significant difference p=0.045 with an unpaired t-test

B) The expression of *ORE1* (AT5G39610) and its *in vivo*-verified targets (Supplemental Table1) from various published transcriptome surveys of hypoxia-treated plants. Expression is calculated relative to samples in normoxia. Not all targets were detected in each dataset, only the detected ones are shown.

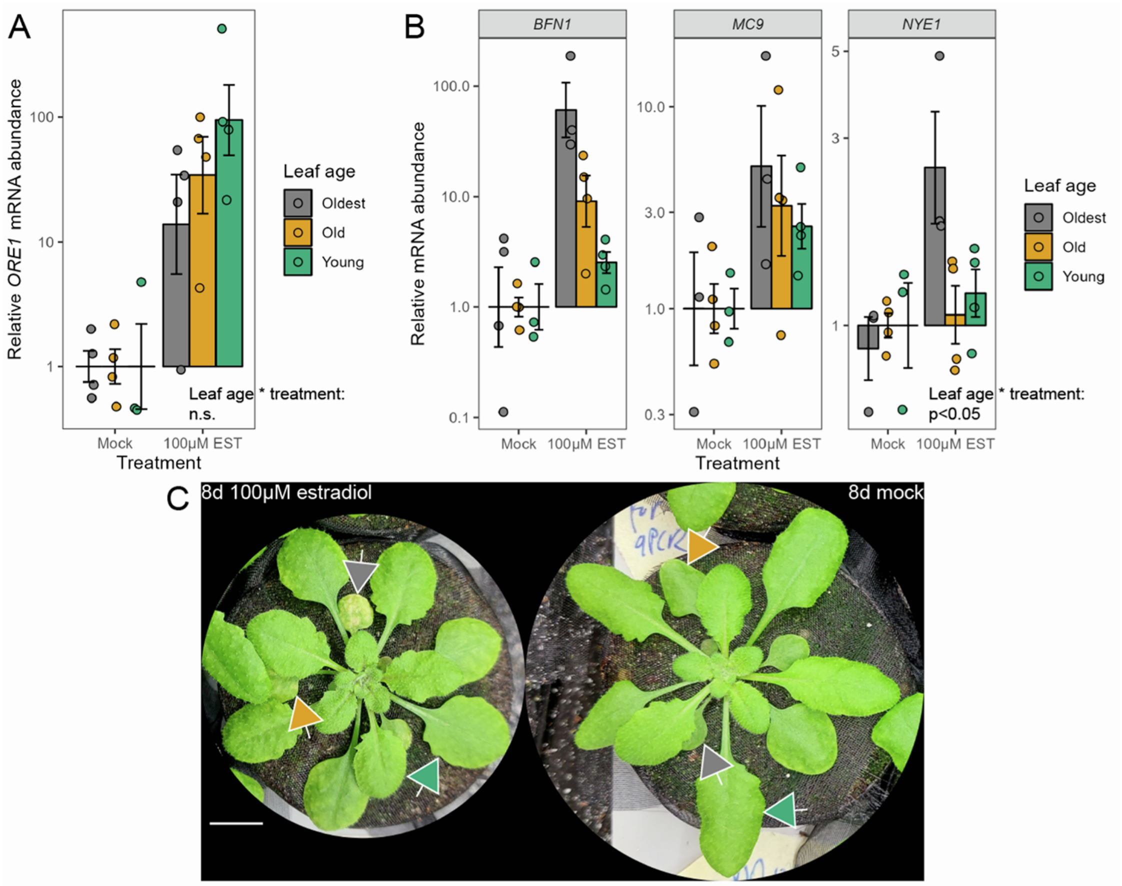

Figure S6

A) mRNA abundance of ORE1 in the oldest leaf (number 1), an old leaf (leaf 3), and a young leaf (leaf 7) of RPS5a::XVE>>ORE1-GFP plants after 8 days of estradiol or mock treatment. n=3-4 per sample, each consisting of 2 leaves of different plants pooled together. The indicated p-value is the interaction effect in a two-way ANOVA for the effect of leaf age and the treatment on *ORE1* expression, with the harvested plant as a blocking effect.

B) mRNA abundance of ORE1 targets BFN1, MC9, and NYE1 in the oldest leaf (number 1), an old leaf (leaf 3), and a young leaf (leaf 7) RPS5a::XVE>>ORE1-GFP plants after 8 days of estradiol or mock treatment. n=3-4 per sample, each consisting of 2 leaves of different plants pooled together. The indicated p-value is the interaction effect in a two-way ANOVA for the effect of leaf age and the treatment on the expression of ORE1 target genes, with the harvested plant and the specific *ORE1* target gene as a blocking effect.

C) Phenotype of RPS5a: XVE>>ORE1-GFP plants after 8 days of estradiol or mock treatment. Arrowheads indicate the leaves harvested in panels A and B, colors correspond to the colors in the legends of these panels. Scale bar indicates 1cm.

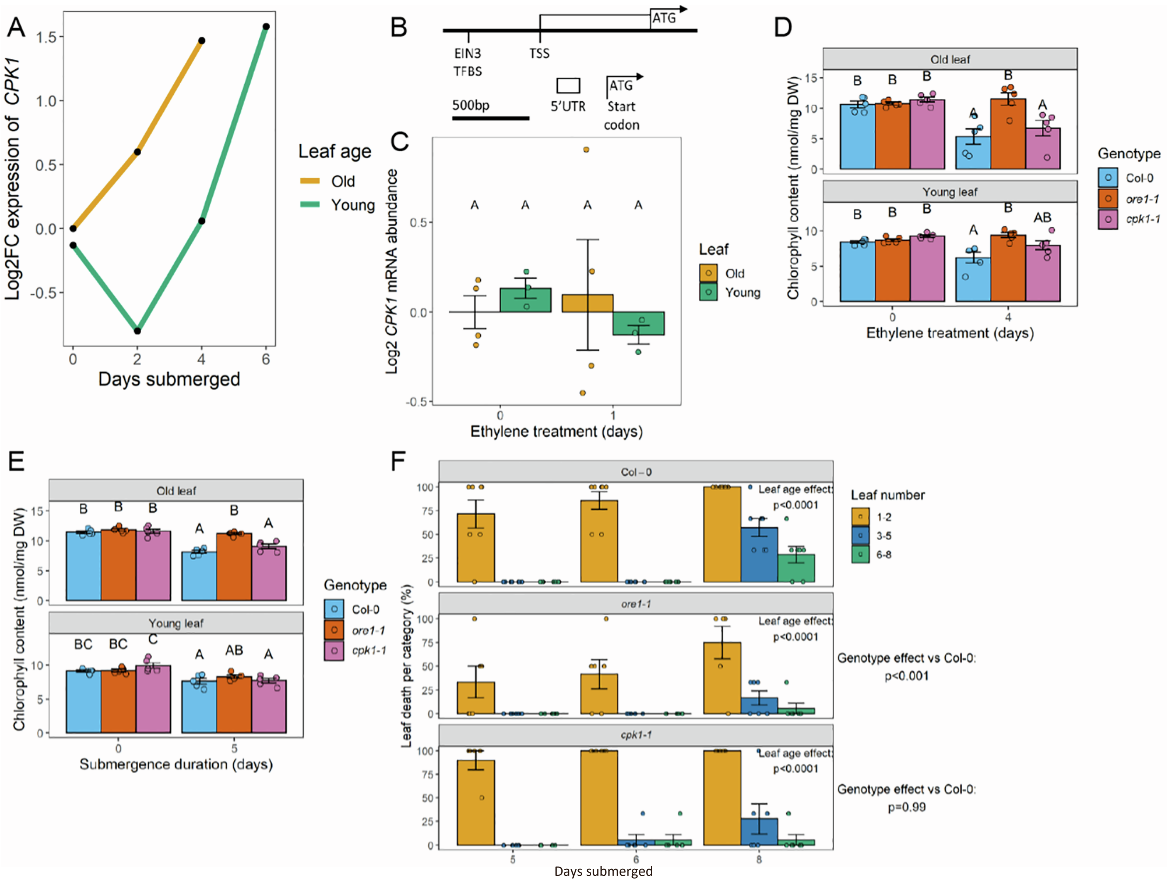

Figure S7

A) Expression of *CPK1* during submergence in old and young leaves, based on RNAseq data. Expression is calculated relative to that of non-submerged old leaves.

B) Schematic overview of the EIN3 binding site (AYGWAYCT)in the promoter of *CPK1,* 443 bp from its transcriptional start site.

C) Expression of *CPK1* in response to ethylene in darkness, as determined by qPCR. Different letters indicate significant differences between groups (two-way ANOVA + Tukey's post-hoc test).n=4 for old leaves; n=3 for young leaves. Two leaves were pooled together per sample.

D-E) Chlorophyll content of Col-0, *ore1-1* and *cpk1-1* plants before and after 5 days of submergence treatment in darkness (D) or 4 days of ethylene treatment in darkness €. Different letters indicate significant differnces between groups (two-way ANOVA + Tukey's post-hoc test), n=5 per sample.

F) Col-0, *ore1-1* and *cpk1-1* plants all show age-dependent leaf death. This process is slowed down in *ore1-1* but not in *cpk1-1*. The leaf age effect was determined by two-way ANOVA (leaf age *time). The genotype effect was determined by two-way ANOVA (genotype+leaf age) followed by Tukey's post-hoc test. p-values correspond to the comparison between either mutant and Col-0 (wild type).

Error bars indicate SEM.

Supplemental Video 1: Time-lapse showing age-dependent leaf death in a representative Arabidopsis (Col-0) plant that was subjected to complete submergence (dark).

Supplemental Video 2: Time-lapse showing differences in the speed of age-dependent leaf death in representative Arabidopsis Col-0 and *ore1-1* genotypes subjected to complete submergence (dark).
